# Supplementary material for: A biomimetic laminated strategy enabled strain-interference free and durable flexible thermistor electronics
Source: Nat Commun. 2022 Oct 29;13:6472. doi: 10.1038/s41467-022-34168-x (PMC9617538; doi:10.1038/s41467-022-34168-x)
Supplement: Supplementary file 1 — Supplementry Information [file 41467_2022_34168_MOESM1_ESM.pdf]

Supporting Information

A Biomimetic Laminated Strategy Enabled Strain-interference Free and Durable Flexible Thermistor Electronics

Sanwei Hao[1], Qingjin Fu[1], Lei Meng[1], Feng Xu[1], and Jun Yang[1,2]*

[1]Beijing Key Laboratory of Lignocellulosic Chemistry, College of Materials Science and Technology, Beijing Forestry University, Beijing 100083, China.

[2]State Key Laboratory of Pulp and Paper Engineering, South China University of Technology, Guangzhou 510640, China.

*E-mail: yangjun11@bjfu.edu.cn Tel: 86-10-62337223.

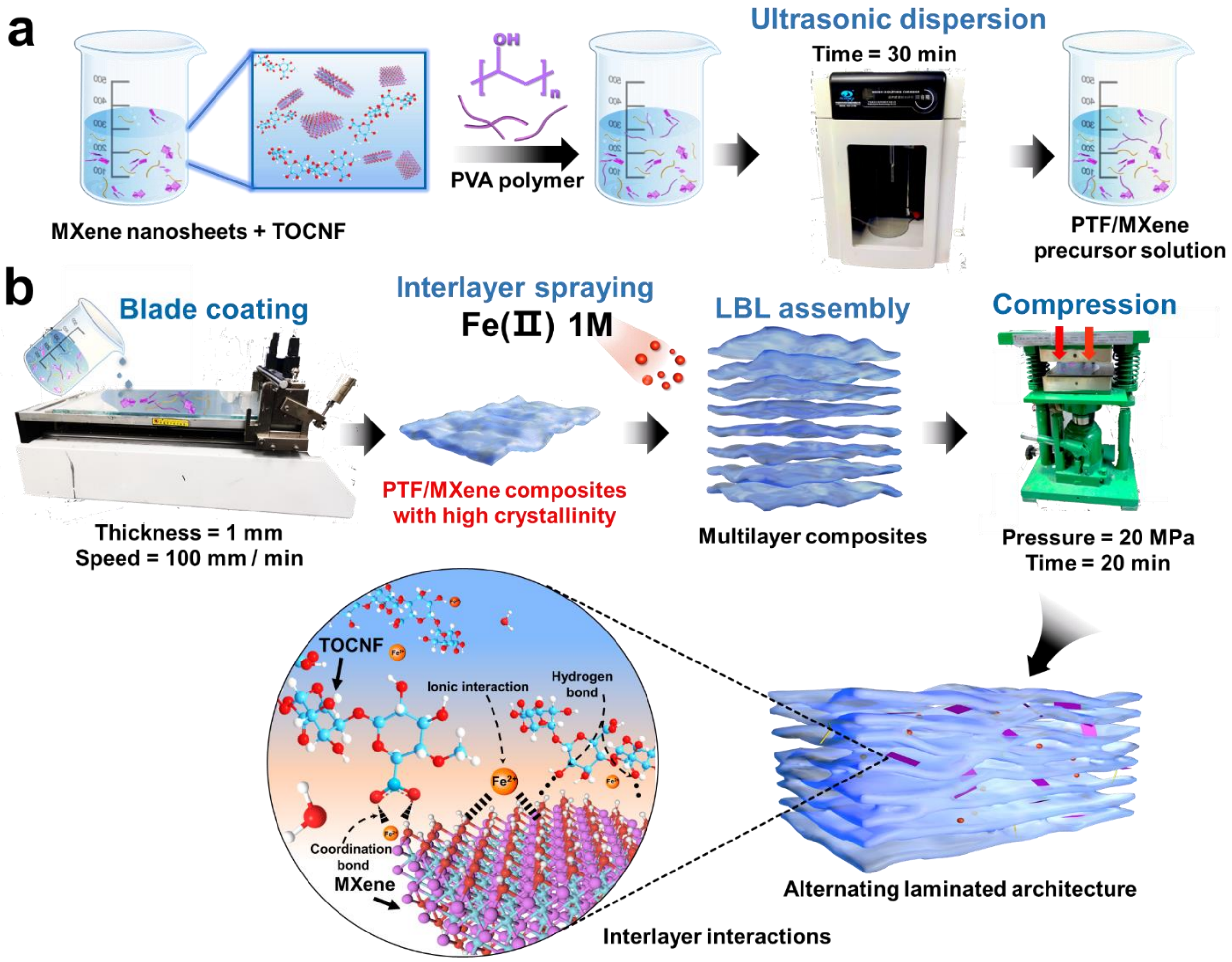

Supplementary Fig. 1. Schematic illustration for preparing the alternating laminated architecture of the PTF/MXene/Fe composites. a Preparation diagram of PTF/MXene precursor solution. **b** The fabrication process and the interfacial bridging of the PTF/MXene/Fe composites.

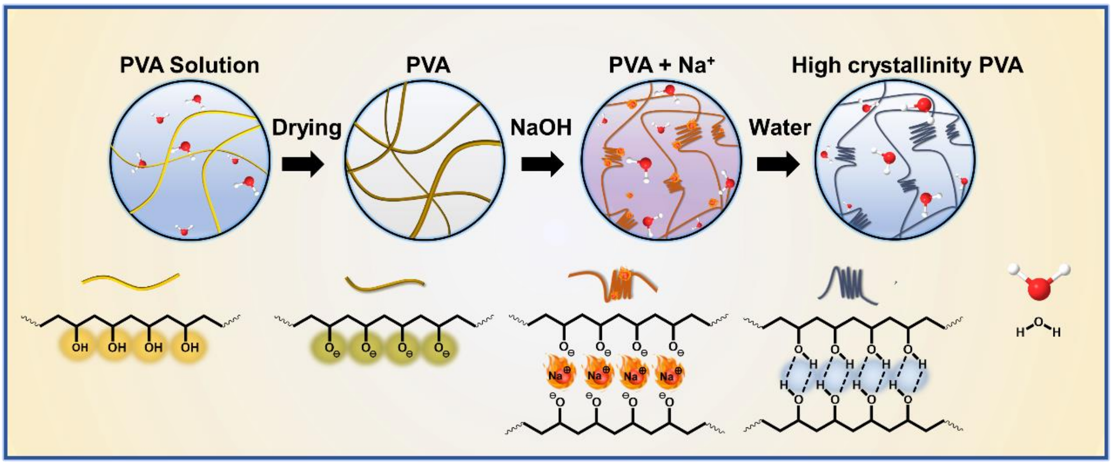

Supplementary Fig. 2. The preparation and proposed mechanism of high crystallinity PVA polymer networks.

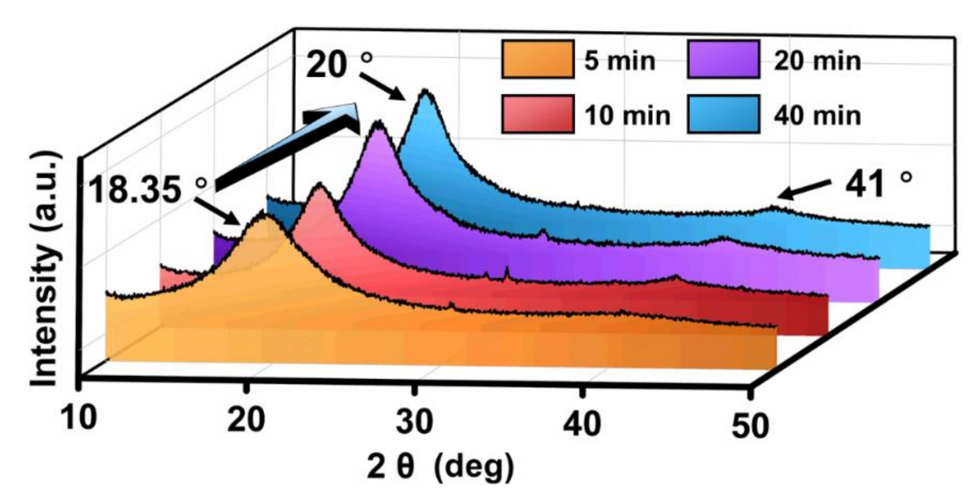

Supplementary Fig. 3. Representative XRD profiles of PVA.

The PVA show strong diffraction peaks at about 18.35°, corresponding to the typical reflection crystal plane of (10$\bar{1}$). The red shift phenomenon occurred with the extension of the immersing time, which was mainly ascribed to the self-association of —OH (PVA) during the metal complexation by Na^+ ions. Additionally, the gradually increased peak intensity of the diffraction profiles indicated the enhancement of crystallinity. Considering that the increasement in peak of crystallinity slowed down after soaking for 20 min, we thereby chose 20 min as a typical soaking time.

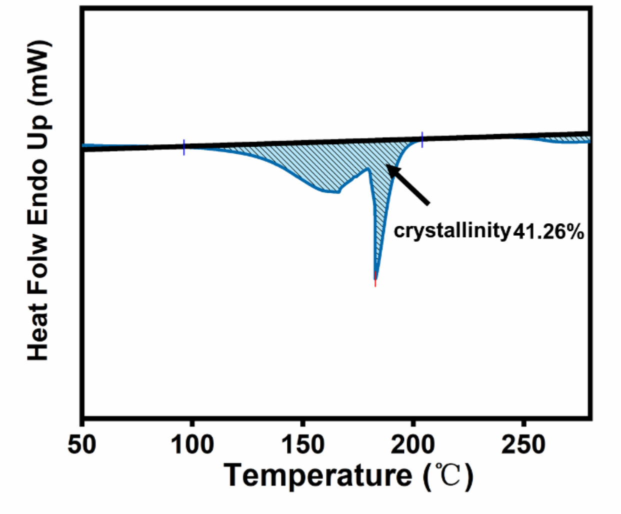

Supplementary Fig. 4. The differential scanning calorimetry (DSC) curve for calculating the crystalline fraction of PVA.

An increased in crystallinity of PVA after NaOH solution immersion was also examined from differential scanning calorimetry (DSC), confirming the rationality of our procedure. The crystallinity of the PVA after NaOH immersion was about 41.26 %, calculated as Eq.S2:

$$\overline{X_c} = \frac{\Delta H_f(T_m)}{\Delta H_f^0(T_m^0)} \quad \text{(S2)}$$

where $\Delta H_f(T_m)$ was the enthalpy fusion at the melting point T_m as measured by DSC. $\Delta H_f^0(T_m^0)$=138.60 $J. g^{-1}$ was the enthalpy fusion of the 100 % crystalline PVA.[7]

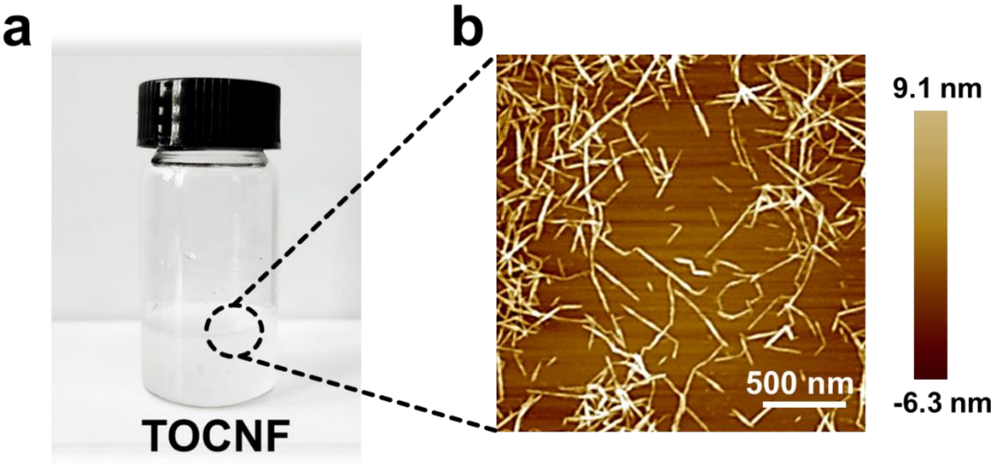

Supplementary Fig. 5. Characterization of TOCNF. a The optical image of TEMPO oxidated cellulose nanofibrils (TOCNF) and **b** the corresponding AFM image showed the size of the obtained TOCNF about 0.8–2.0 um and several nanometers in length and diameter, respectively.

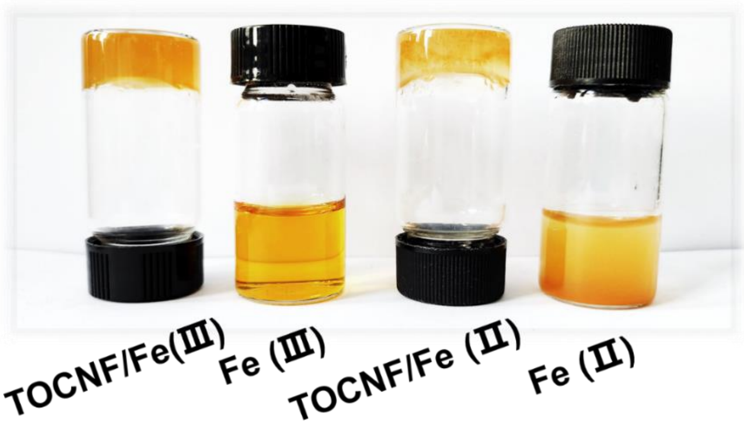

Supplementary Fig. 6. The photos of TOCNF cross-linked by Fe (II) and Fe (III).

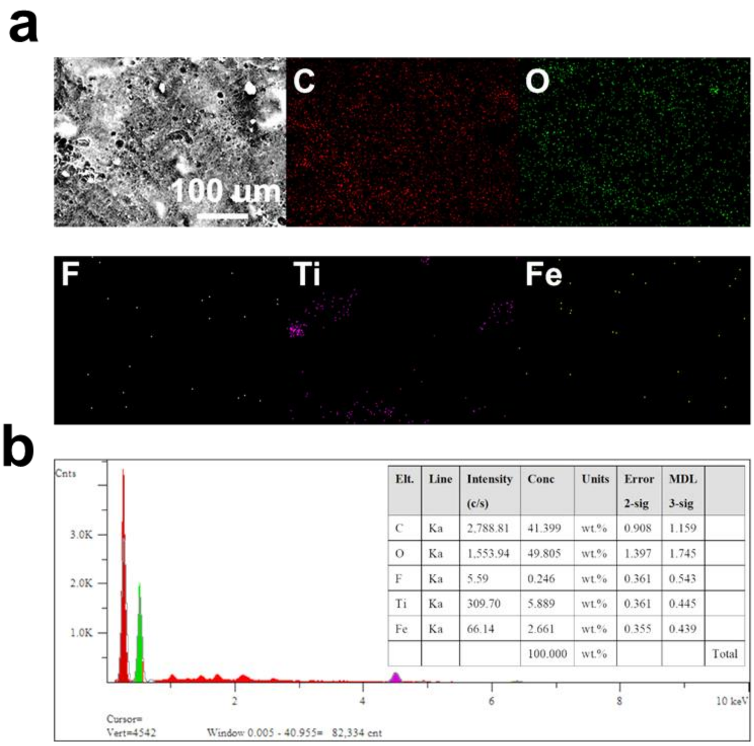

Elt.	Line	Intensity (c/s)	Conc	Units	Error 2-sig	MDL 3-sig	
C	Ka	2,788.81	41.399	wt.%	0.908	1.159	
O	Ka	1,553.94	49.805	wt.%	1.397	1.745	
F	Ka	5.59	0.246	wt.%	0.361	0.543	
Ti	Ka	309.70	5.889	wt.%	0.361	0.445	
Fe	Ka	66.14	2.661	wt.%	0.355	0.439	
			100.000	wt.%			Total

Supplementary Fig. 7. SEM images and element analysis. a SEM images of PTF/MXene/Fe membrane and **b** the corresponding EDS spectra of the selected area.

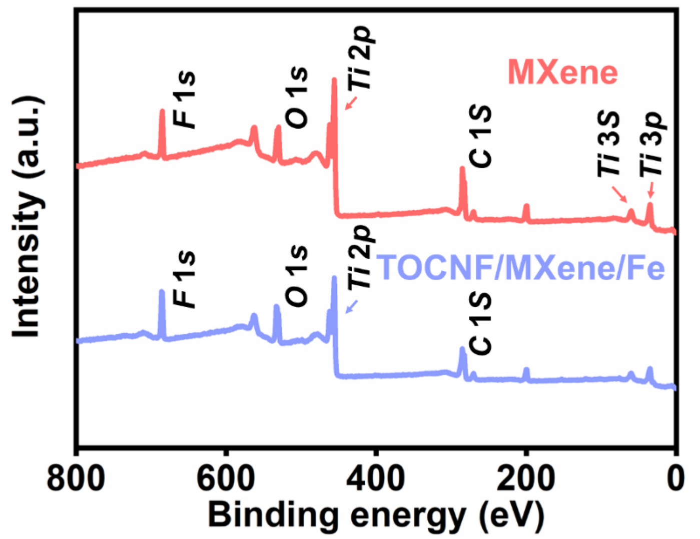

Supplementary Fig. 8. XPS survey spectrum of the MXene and TOCNF/MXene/Fe.

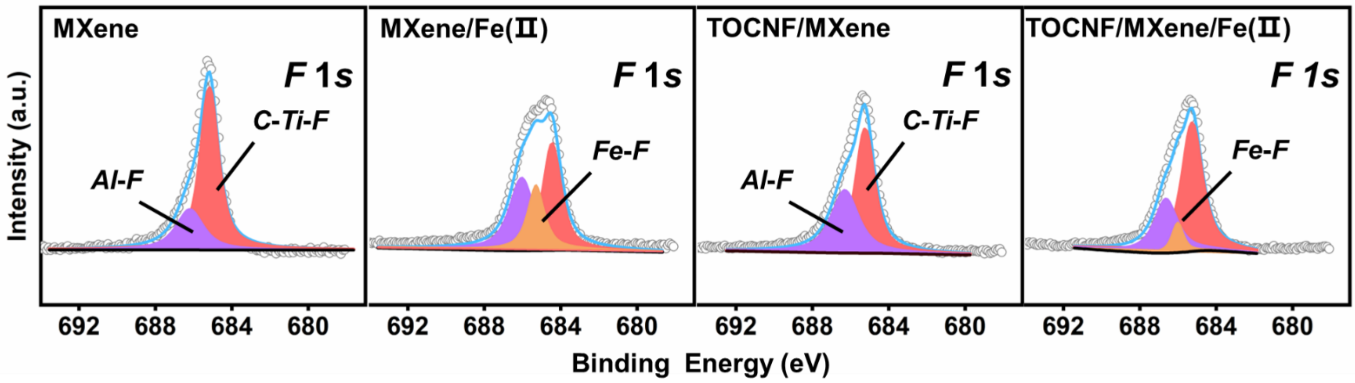

Supplementary Fig. 9. *F* 1*s* XPS spectra of MXene, MXene/Fe, TOCNF/MXene, and TOCNF/MXene/Fe, respectively.

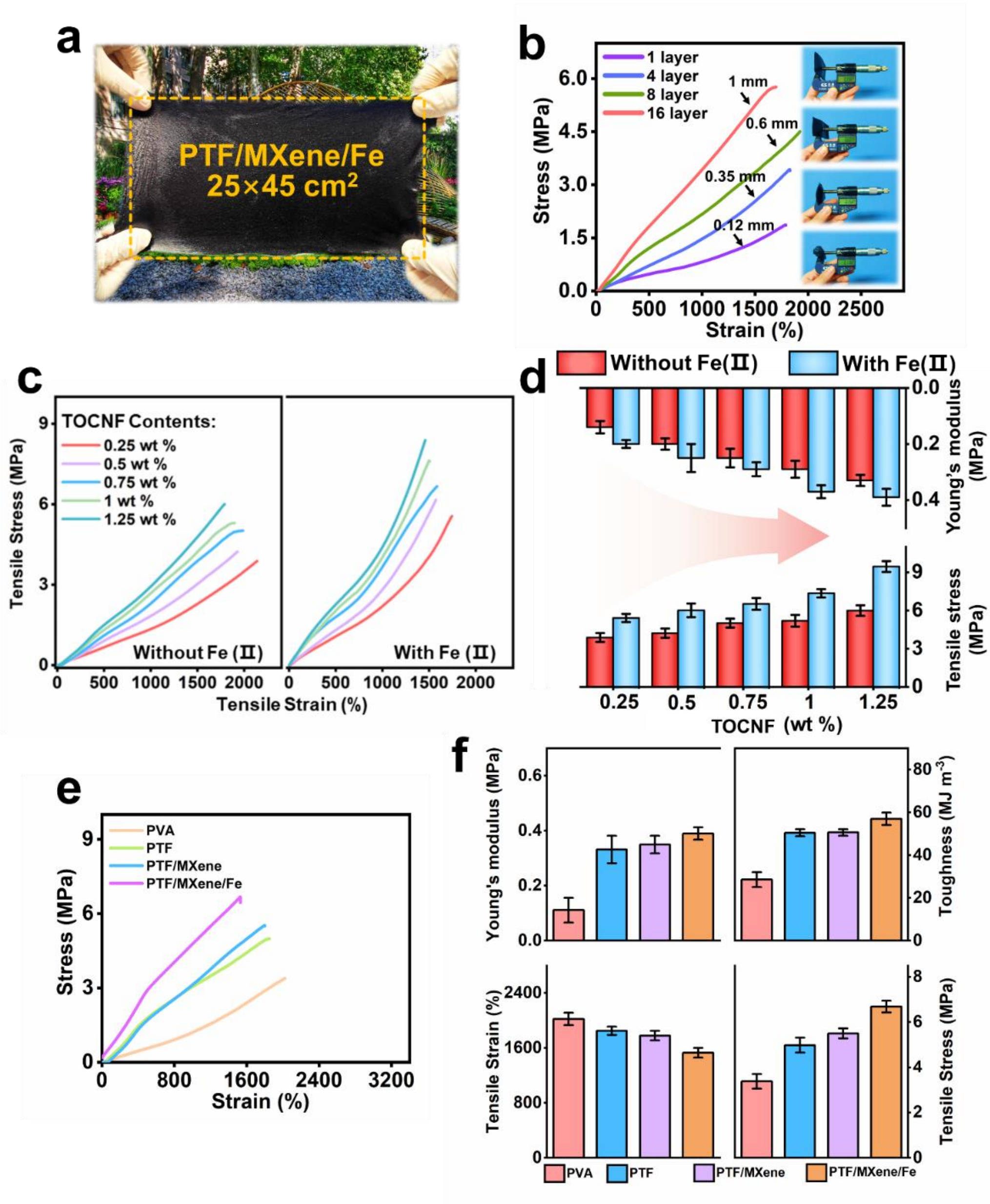

Supplementary Fig. 10. Mechanical optimization. **a** Optical photograph of the resultant large-area (25 × 45 cm^2) PTF/MXene/Fe composites by LBL assembly, showing favorable scalability. **b** The laminated layer number (1, 4, 8, and 16 layers, respectively) dependent tensile stress–strain curves of pristine PVA with alternating laminated architecture. **c** The typical tensile stress–strain curves of PTF/MXene and PTF/MXene/Fe composites with different TOCNF contents (0.25, 0.5, 0.75, 1, and 1.25 wt%, respectively), and **d** the corresponding TOCNF content dependent Young's modulus and tensile stress. **e** Uniaxial tensile test of PVA, PTF, PTF/MXene, and PTF/MXene/Fe composites, respectively, and **f** the corresponding mechanical parameters including Young's modulus, toughness, tensile strain, and tensile stress. Data in **d** and **f** are presented as mean values ± SD, n = 3 independent composites.

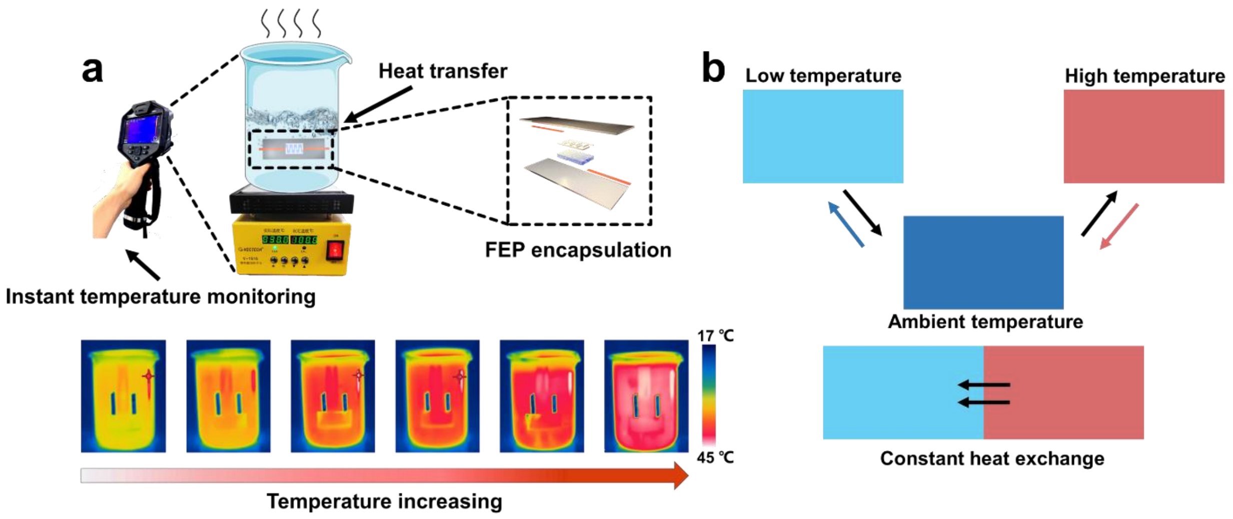

Supplementary Fig. 11. Schematic diagram of thermosensation measurement. a The water as the heat transfer medium. **b** Scheme of thermal diffusion effect in continuous temperature measurement.

Notably, the thermal diffusion effect will cause continuous environmental heat exchange, thus the offered constant temperature conditions are not supported the long-term temperature cyclic tests.

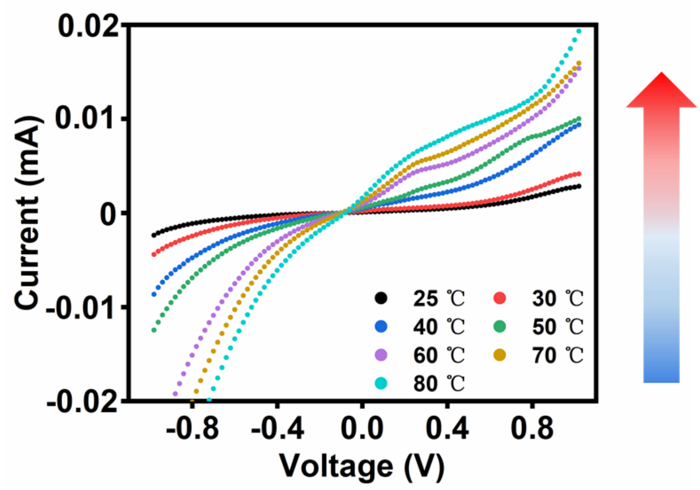

Supplementary Fig. 12. ***I*−*V* curves of the TES under different temperatures ranging from 25 to 70 °C.**

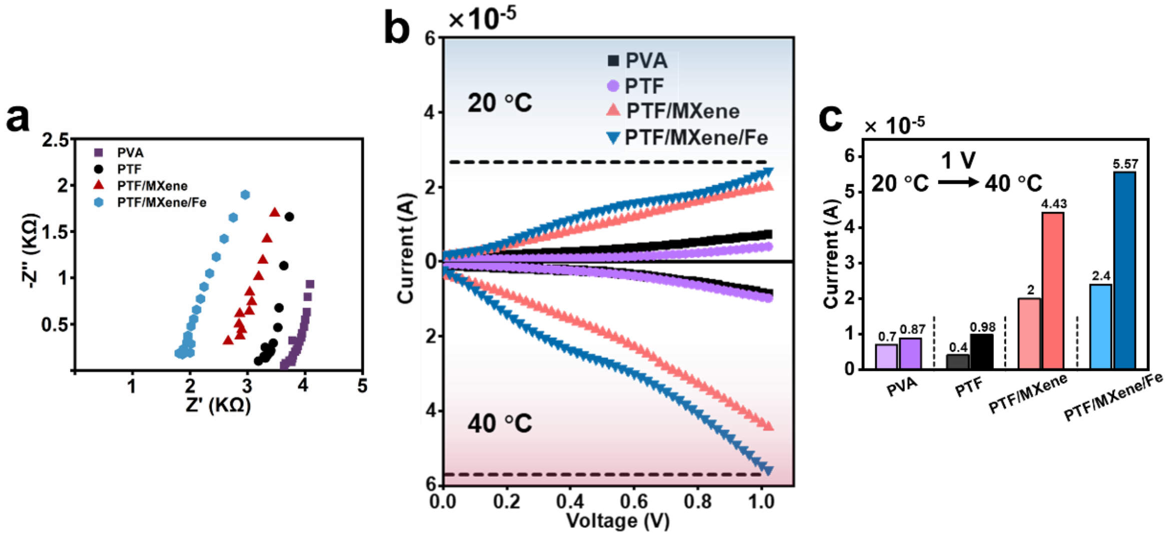

Supplementary Fig. 13. Electrochemical performances rely on MXene nanosheets. **a** The electrochemical impedance spectroscopy (EIS) of PVA, PTF, PTF/MXene, and PTF/MXene/Fe composites, respectively, and **b** measured *I*–*V* curves of these composites at 20 °C and 40 °C. **c** Comparison of current variations at 1 V from 20 to 40 °C for PVA, PTF, PTF/MXene, and PTF/MXene/Fe composites, respectively.

The electrochemical properties of PVA, PTF, PTF/MXene, and PTF/MXene/Fe composites are characterized by electrochemical impedance spectroscopy (EIS) where the intercepts of EIS curves with x axis can be considered as the impedance. It is observed that the PTF/MXene/Fe composites possess the lowest impedance (Supplementary Fig. 13a), evidencing that the MXene nanosheets played the key role in electrochemical performances. Supplementary Fig. 13b and c further present the I-V curves and current values at 1 V of PVA, PTF, PTF/MXene, and PTF/MXene/Fe composites at 20 °C and 40 °C, where PVA and PTF composites remain virtually constant. In contrast, the current values of PTF/MXene, and PTF/MXene/Fe composites dramatically increase (from 2 to 4.43, and 2.4 to 5.57, respectively), which is consistent with the increasing tendency of temperature. These findings further suggest that the thermosensation mainly ascribed to MXene nanosheets.

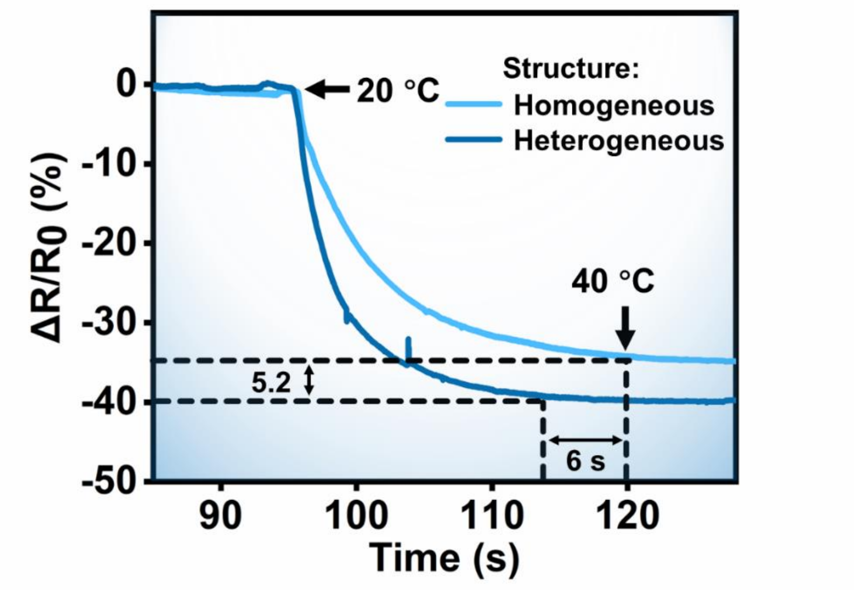

Supplementary Fig. 14. Comparison of temperature response for TES with homogeneous elastomer structure and interlocking laminated architecture from 20 to 40 °C.

We fabricate and examine a type of reference sensor, the homogenous elastomer sensor, which keeps the TOCNF, MXene nanosheets, and Fe contents, only eliminating layer-by-layer assembly and interlayer spraying processes. As a result, the homogeneous elastomer sensor shows resistance variation (~ 34.2) associated with response time (~ 20 s) from 20 to 40 °C, while the TES in conjunction with LBL assembly that facilitating MXene nanosheets forming thermal paths possesses obvious variation value about 39.4 with fast response time (~ 13.5 s). This phenomenon reflects that the tight packing of MXene nanosheets during the LBL process may facilitate the construction of thermally conductive pathways and reduce the energy barriers for electron hopping.

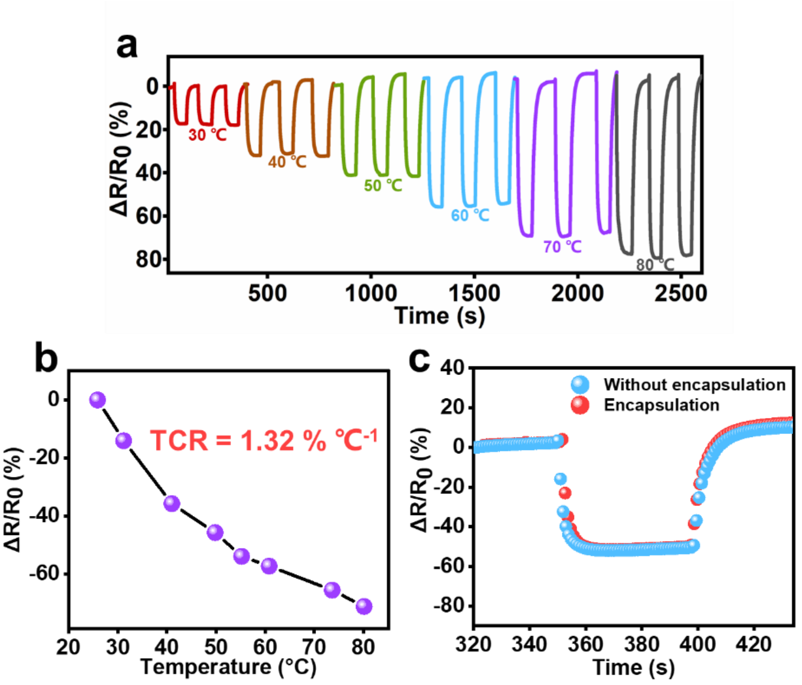

Supplementary Fig. 15. The stable thermosensation of TES. a Temperature-dependent relative resistance curves of TES by different batches and **b** the corresponding temperature coefficient of resistance (TCR). **c** The neglectable heat transfer obstruction of the ultrathin FEP encapsulation layer (80 um) for stable thermal performances of TES.

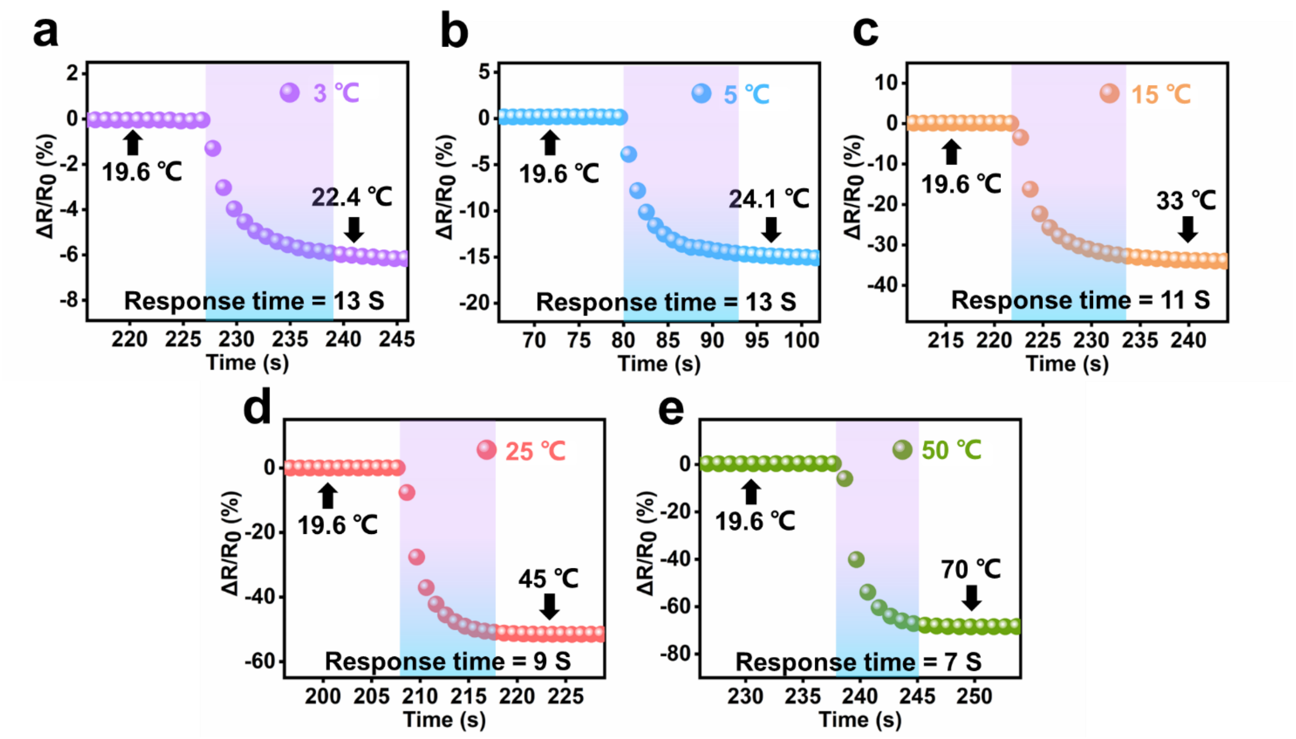

Supplementary Fig. 16. Thermal response time. The rapid thermosensation of TES to different temperature gradients from constant initial 19.6 ℃ to **a** 22.4 ℃, **b** 24.1℃, **c** 33 ℃, **d** 45 ℃, and **e** 70 ℃, respectively).

Notably, the thermal resistance variations at 22.4 °C (13 s) required a much longer time than that of at 70 °C (7 s). We attributed this result to the fact that a larger temperature gradient between the heat source (70 °C) and ambient surrounding (19.6 °C) may cause a stronger thermal convection, resulting in more dramatic resistance changes and achieved the heat equilibrium in a shorter time.

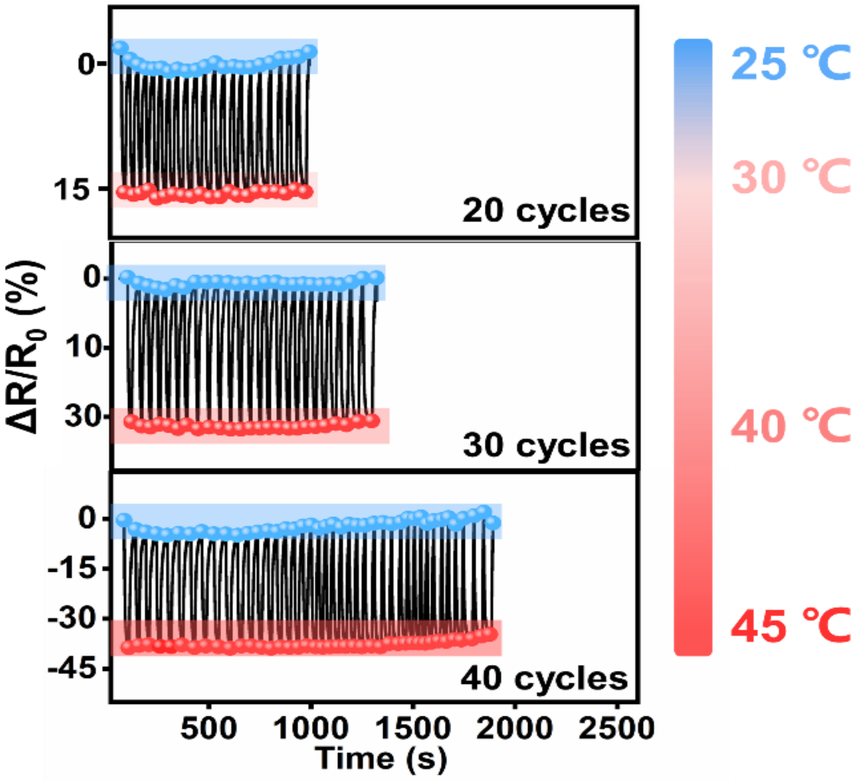

Supplementary Fig. 17. Reproducible temperature discrimination during various repetitive heating-cooling cycles (25-30 °C, 25-40 °C, and 25-45 °C).

The relative resistance variation displayed distinct divergence between cold-hot temperature interval, and the output signals under each temperature gradient showed negligible deviation, implying high durability and stability of the TES sensors.

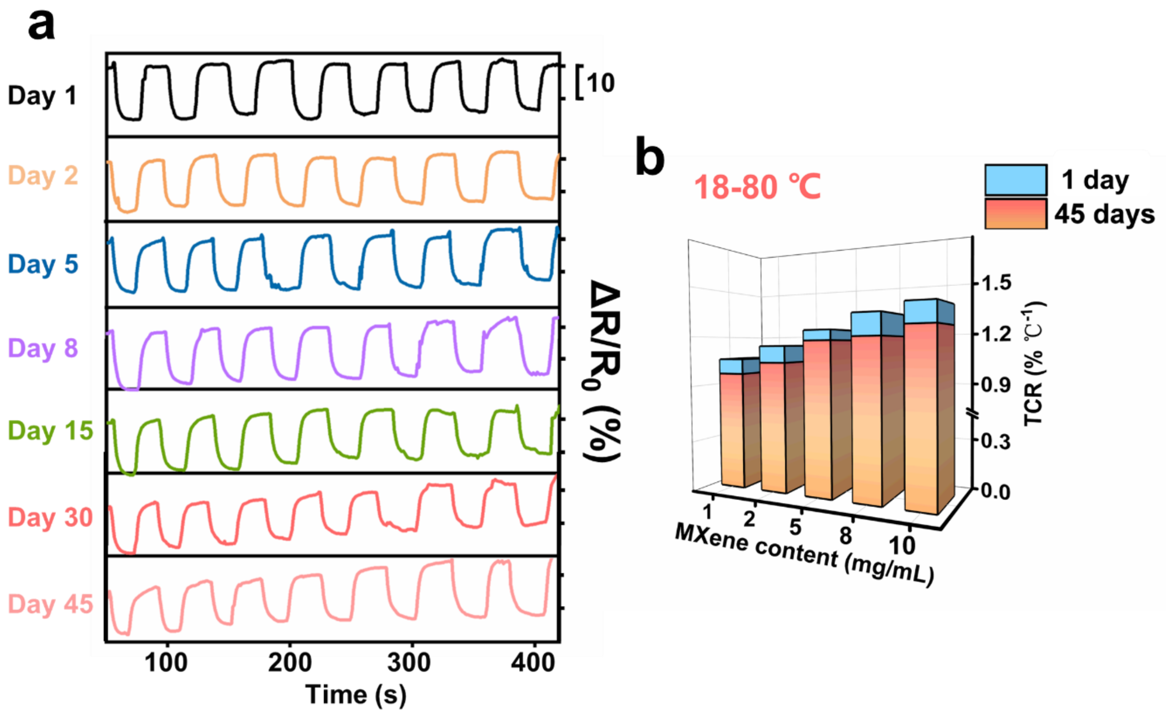

Supplementary Fig. 18. Long life-span of TES. a Long-term operation of the TES over 45 days in the body temperature range of 30-40 °C. **b** The subtle TCR variation of TES with different contents of MXene nanosheets over 45 days.

As for the stability concern, we also investigate the time gradient influence on the reliability of TES, and the stable signal output still could be recorded and possesses 90.9% TCR value retention even over a long period of 45 days that sufficient for the most of possible application duration, corroborating the extraordinary long-term reliability of the TES.

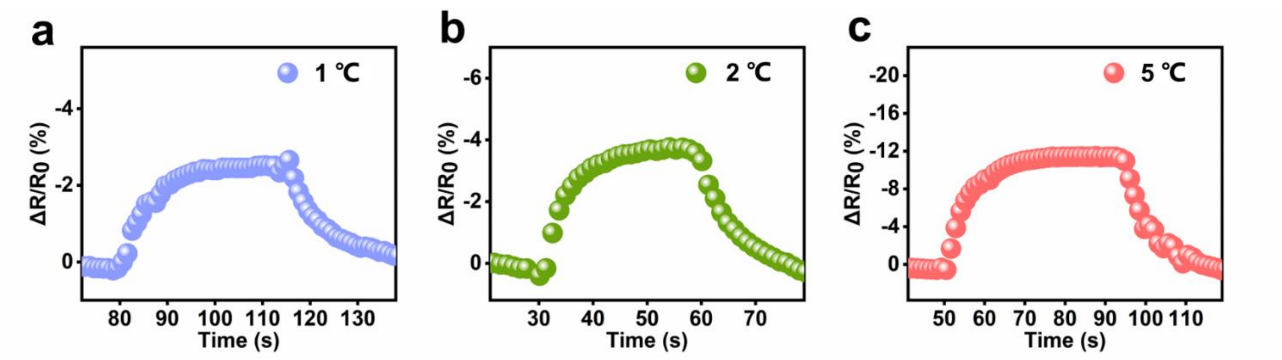

Supplementary Fig. 19. Highly discernible temperature resolution of TES. a $\Delta R/R_0$ of TES under subtle temperature changes (1 °C). **b** $\Delta R/R_0$ of TES under middle temperature changes (2 °C). **c** $\Delta R/R_0$ of TES under high temperature changes (5 °C).

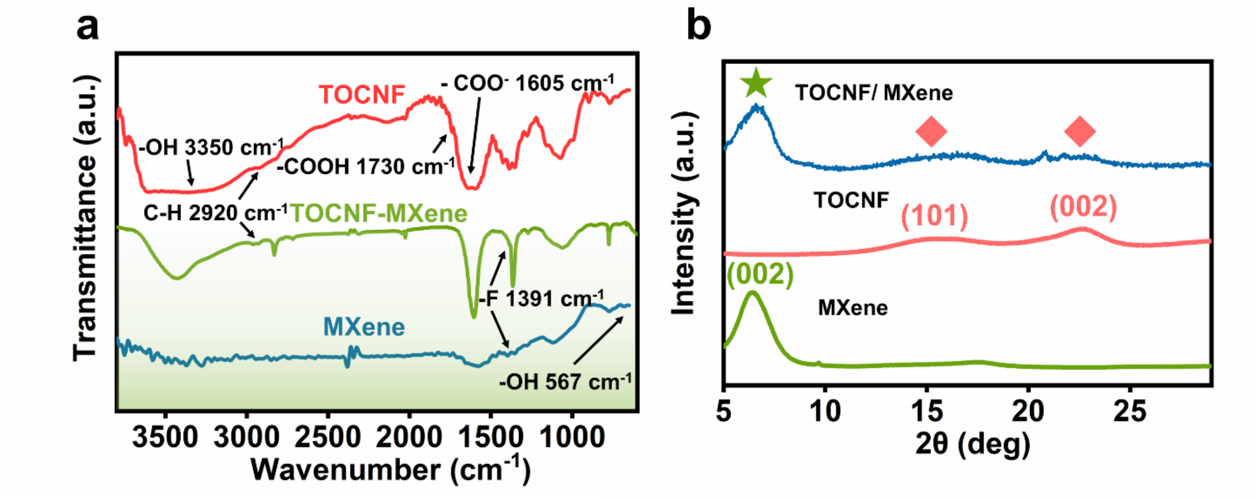

Supplementary Fig. 20. The FTIR spectra and XRD patterns. **a** FTIR spectra and **b** XRD patterns of the $Ti_3C_2T_x$ (MXene), TOCNF, and TOCNF/$Ti_3C_2T_x$ (MXene), respectively.

Notably, the cellulose characteristic absorption bands at 2920 cm^{-1} (C−H stretching) were observed in the FTIR spectrum of the TOCNF/MXene nanocomposites. The XRD patterns showed that the as-prepared TOCNF possesses the typical (101) and $(10\bar{1})$ crystallographic planes of cellulose I crystalline structure at 2θ = 14.0 - 17.8°. Meanwhile, the obtained TOCNF/MXene nanocomposites also showed an obvious peak at 2θ = 22.5° corresponds to the (002) crystal plane of cellulose I crystalline structure, indicating the successful combination of the two components.

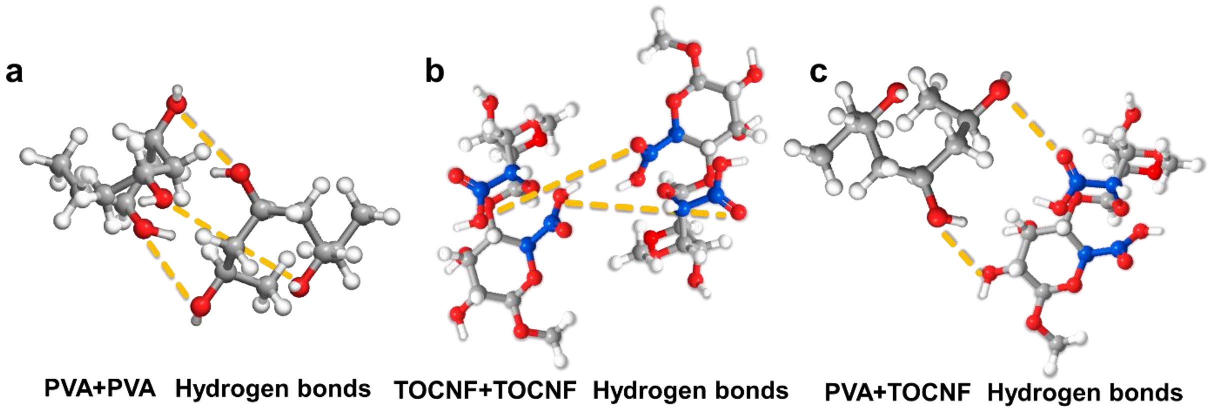

Supplementary Fig. 21. Dynamic cross-linking. The multiple hydrogen bonds in **a** PVA, **b** TOCNF, and **c** TOCNF+PVA, respectively, acting as the dynamic cross-linking for energy dissipation.

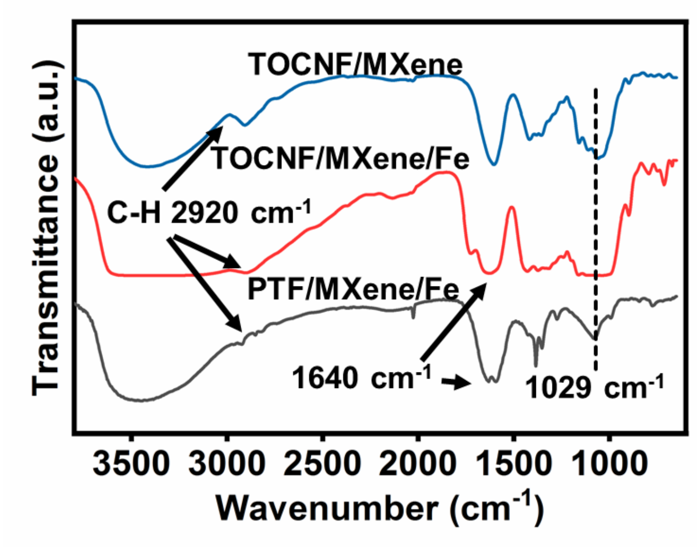

Supplementary Fig. 22. FTIR spectra of TOCNF/MXene, TOCNF/MXene/Fe, and PTF/MXene/Fe, respectively.

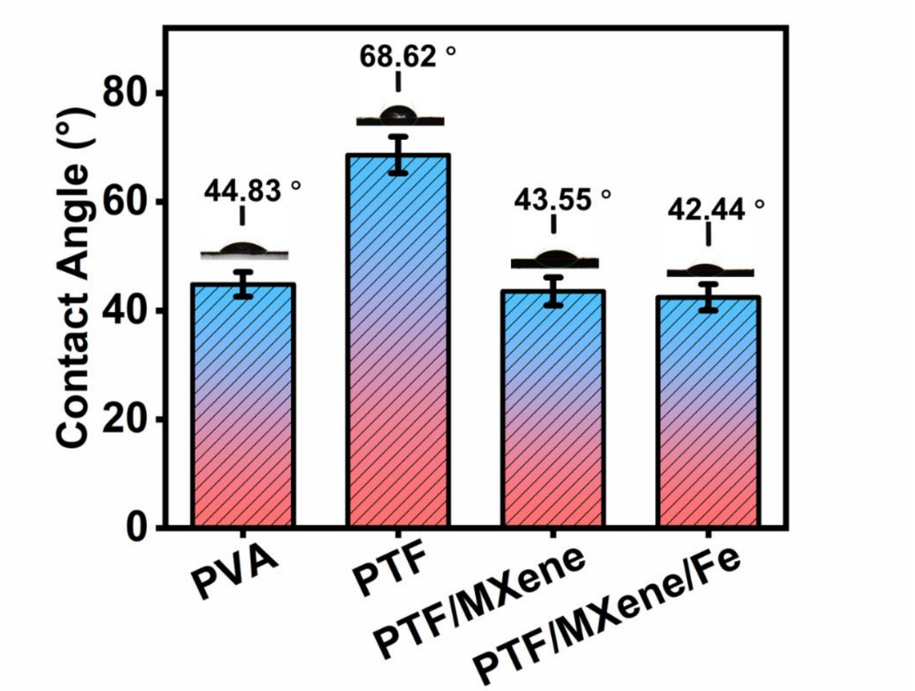

Supplementary Fig. 23. Contact angle measurements for PVA, PTF, PTF/MXene, and PTF/MXene/Fe composites, respectively. Error bars were defined as S.D. (n = 3 independent samples).

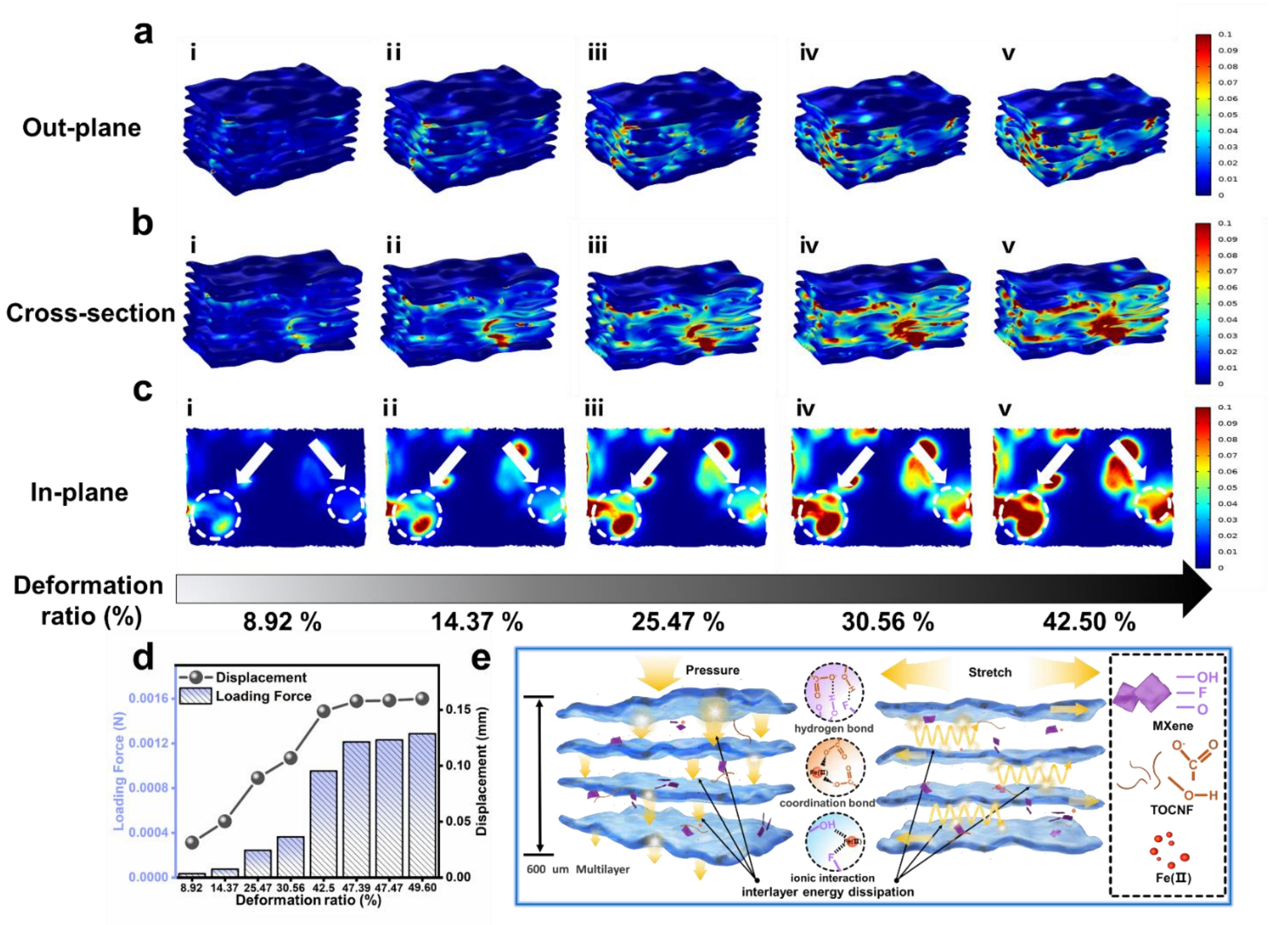

Supplementary Fig. 24. Finite element simulation results. **a** The out-plane view, **b** cross-section view, and **c** in-plane view of stress diffusion at different deformation ratios. **d** Deformation ratio dependent loading force (left axis) and displacement (right axis) in finite element simulation. **e** Schematic diagram of energy dissipation among adjacent layers via multiple hydrogen bonding interactions, coordination bond, and ionic interaction.

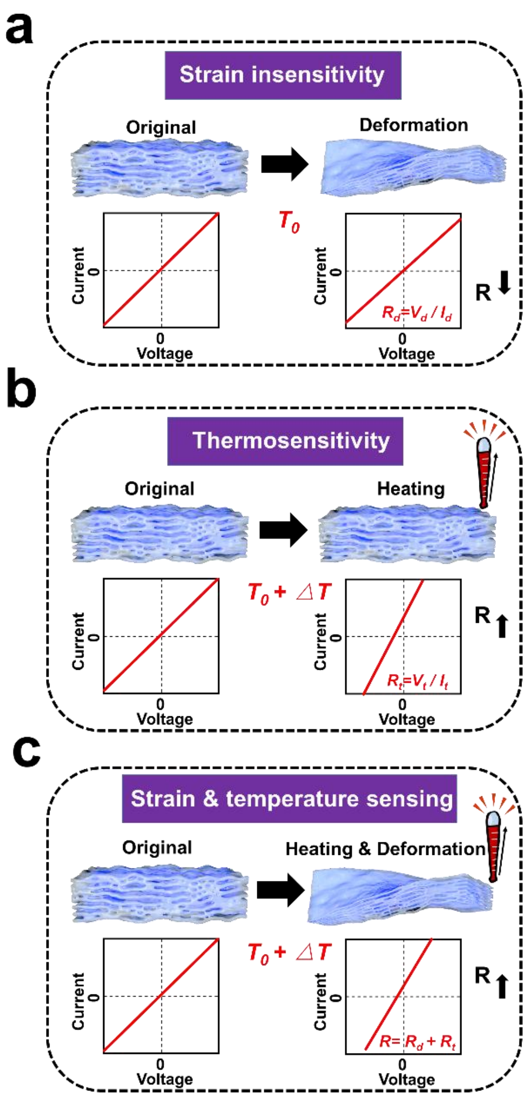

Supplementary Fig. 25. The strain and temperature decoupled mechanism. The Schematic illustration of the bimodal decoupled mechanism for the thermistor sensor when **a** the strain is applied, **b** a temperature gradient is applied, and **c** coupled strain and temperature stimuli are applied simultaneously.

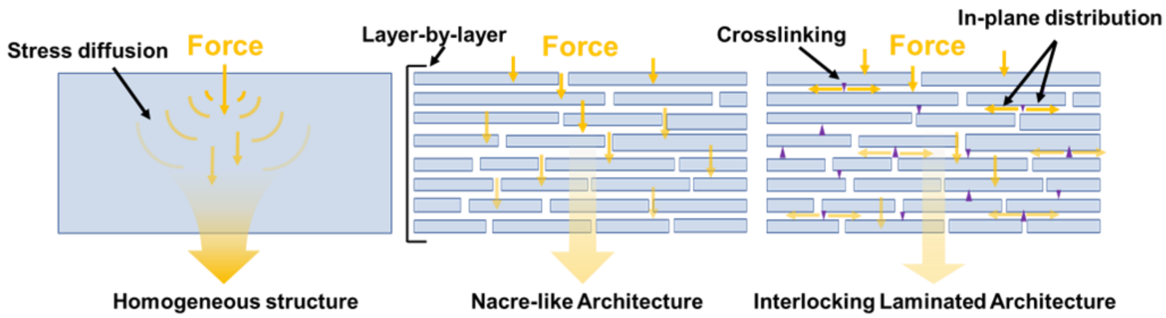

Supplementary Fig. 26. The comparison of stress dispersion under external loading in the homogeneous structure, nacre-mimetic architecture, and interlocking laminated architecture.

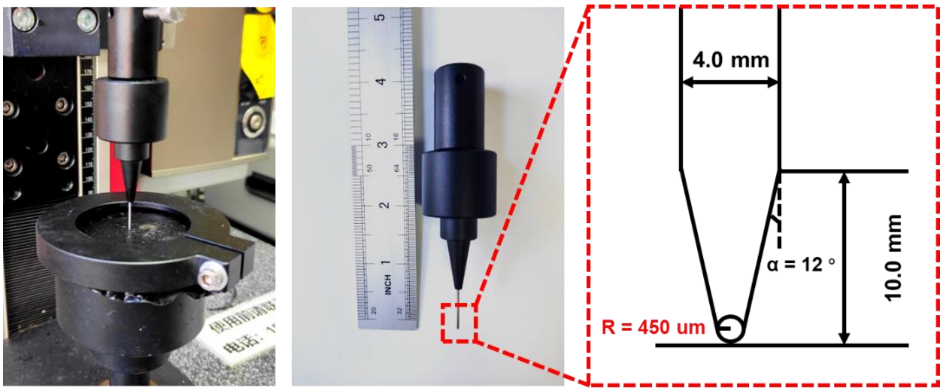

Supplementary Fig. 27. Photographs of puncture resistance test and the corresponding needle size parameters.

Typically, the PTF/MXene/Fe composites of sensing layer (0.60 mm) could withstand a puncture force (needle diameter = 900 um, loading speed = 50 mm min^{-1}) as high as 13.42 N, which is superior to PVA, PTF, and PTF/MXene composites with the force is only 3.9, 6.7, and 7.6 N, respectively. This striking out-plane puncture tolerance further reflects the dominant role of coordination bonds in topological interlocking where the efficient stress dissipation is achieved through the interfacial bridging so as to enable excellent impact resistance.

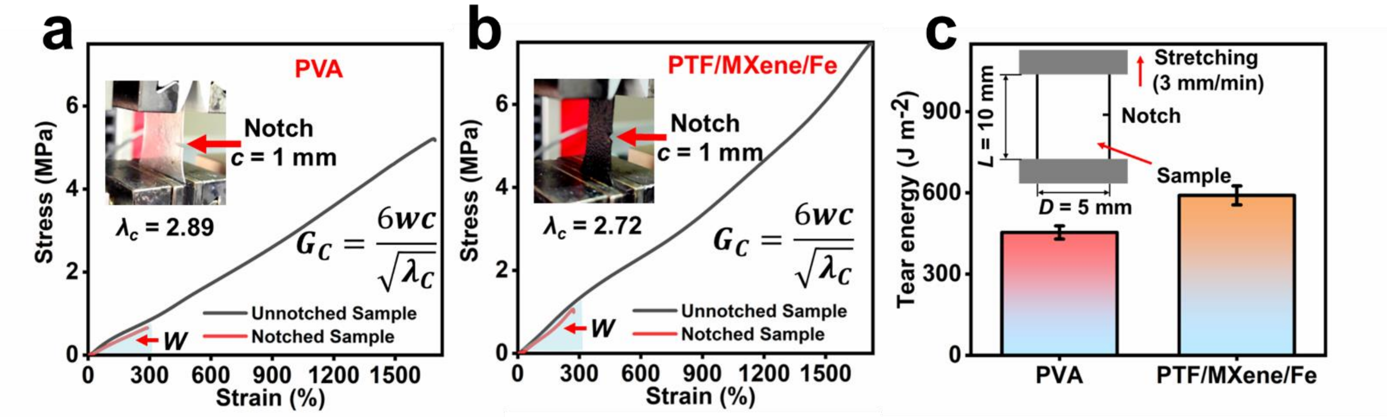

Supplementary Fig. 28. The tearing test. Typical stress–strain curves of the unnotched and notched **a** PVA and **b** PTF/MXene/Fe composites, the inset photograph of the elongation for notched composites and the formula of tearing energy calculation. **c** Tearing energy of PVA and PTF/MXene/Fe composites, error bars were defined as S.D. (n = 3 independent samples) for standard deviations.

The tearing energy test was conducted by the tensile test using the single-edge notched sample (length of the slit = 1 mm). The notched and unnotched specimens (gauge length of 10 mm, width of 5 mm, thickness of 0.6 mm) were both tested at the constant stretching speed of 3 mm min^{-1}. The fracture energy (Gc) was calculated by the Eq.S3:

$$G_C = \frac{6wc}{\sqrt{\lambda_c}} \quad \text{(S3)}$$

where c represented the length of the slit (1 mm), λ_c represented the elongation-

at-break of the notched sample, w represented the strain energy calculated by integration of the stress-strain curve of the unnotched specimen until ε_c ($\varepsilon_c = \lambda_c$ - 1).

With the elongation of notched rectangular PTF/MXene/Fe composites specimen (size = 10×5 mm, stretching speed = 3 mm min^{-1}), the crack gradually widens along the longitudinal direction until the sample becomes damaged at a strain of 1398 %. The fracture energy of the PTF/MXene/Fe composites is calculated to be as large as 591± 35 kJ m^{-2}, which is over 1.3 times higher than that of PVA (454 ± 24 kJ m^{-2}), because of the presence of interfacial interlocking and dynamic cross-linking in the matrix.

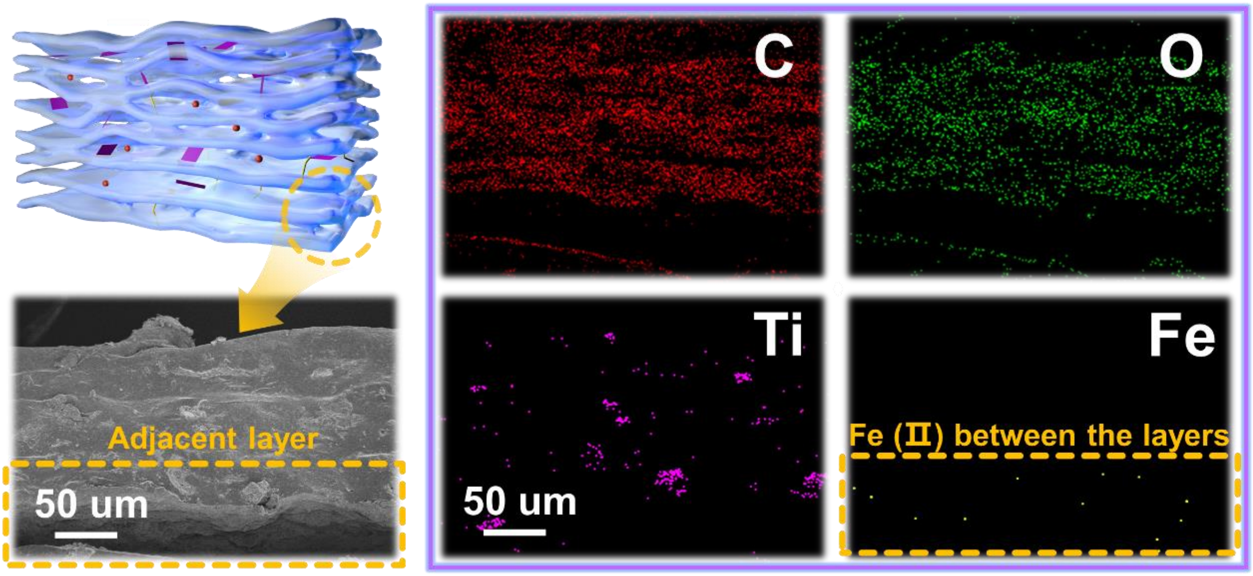

Supplementary Fig. 29. The EDS mapping images for uniform distribution elements of C, O, and Ti, and the detailed spatial distribution of Fe as interfacial bridging agent.

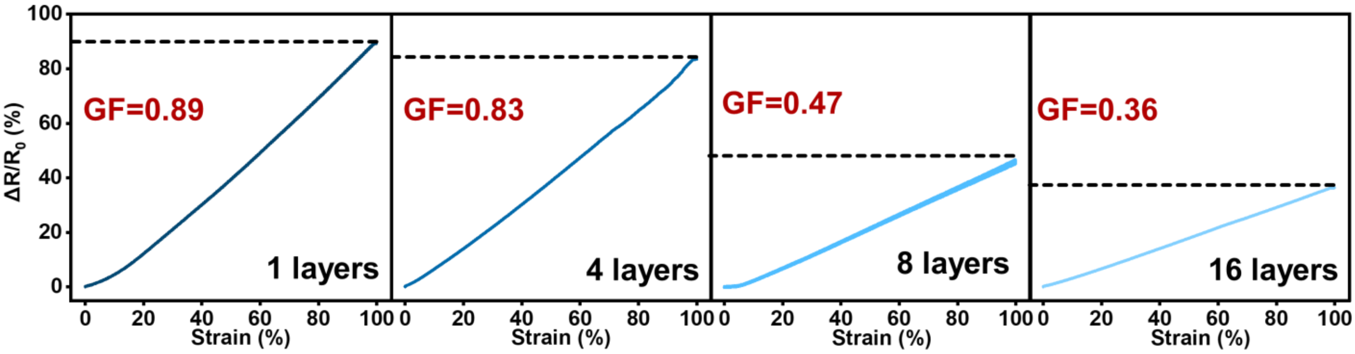

Supplementary Fig. 30. The relative resistance variations and strain sensitivity of TES with different sensing layer number (1, 4, 8, and 16, respectively) under 100 % deformation.

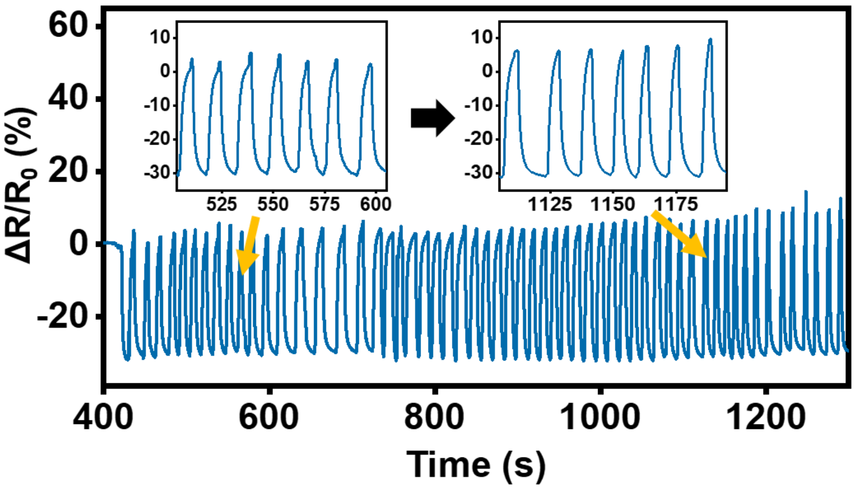

Supplementary Fig. 31. Δ*R*/*R*0 curves of the TES under hairdryer heating flow (54.8 °C) for 70 cycles, showing excellent durability for high temperature monitoring.

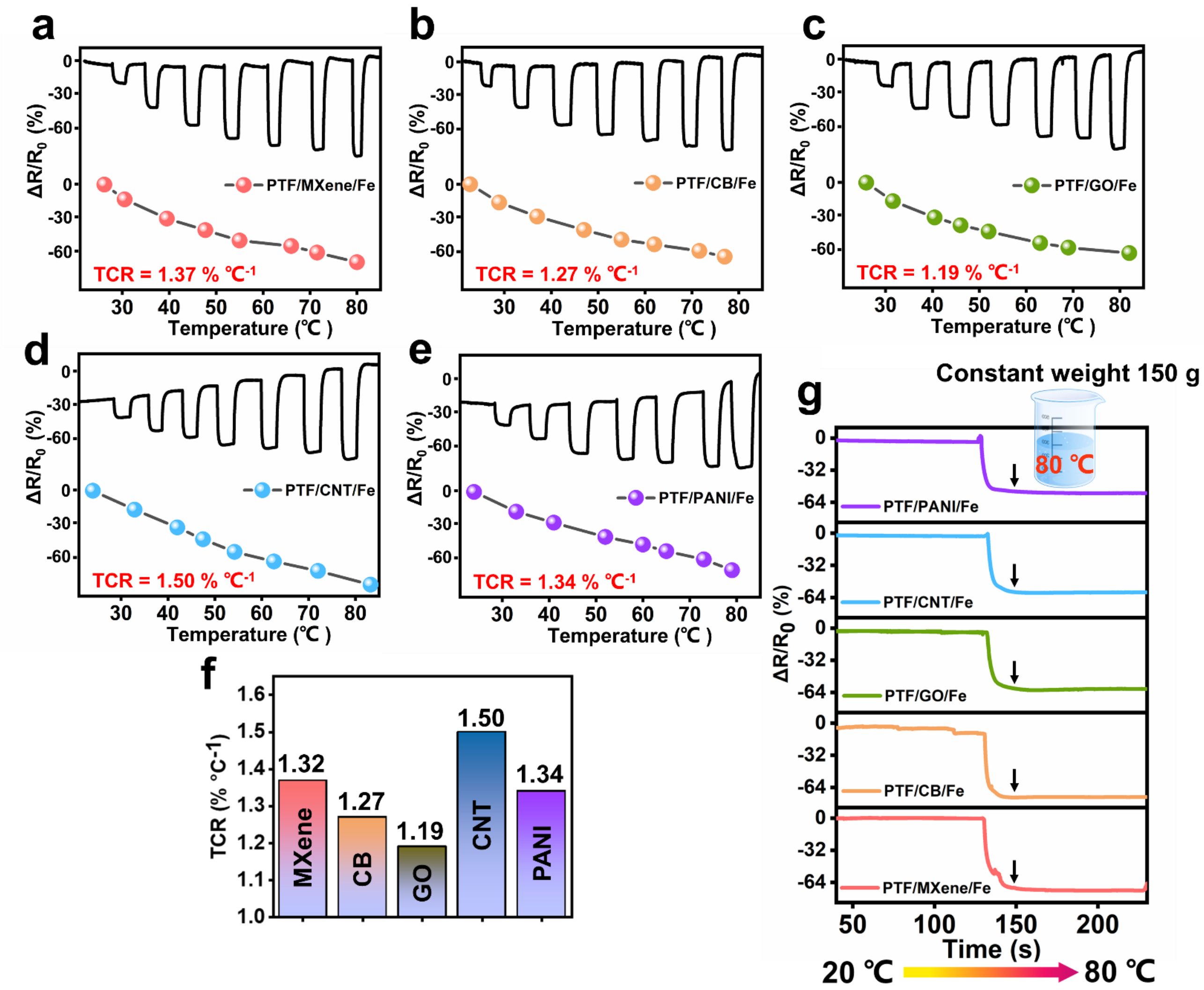

Supplementary Fig. 32. Broad validity of the paradigm of the alternating laminated architecture. The relative resistance variations of TES integrated with various thermosensitive nanofillers including **a** MXene nanosheets, **b** carbon black (CB), **c** graphene (GO), **d** carbon nanotube (CNT), and **e** polyaniline (PANI). **f** The corresponding TCR and (g) temperature monitoring without signal distortion confirmed by simultaneously applying heating (80 ℃) and pressure stimulus (150 g) on the TES integrated with various thermosensitive nanofillers.

.

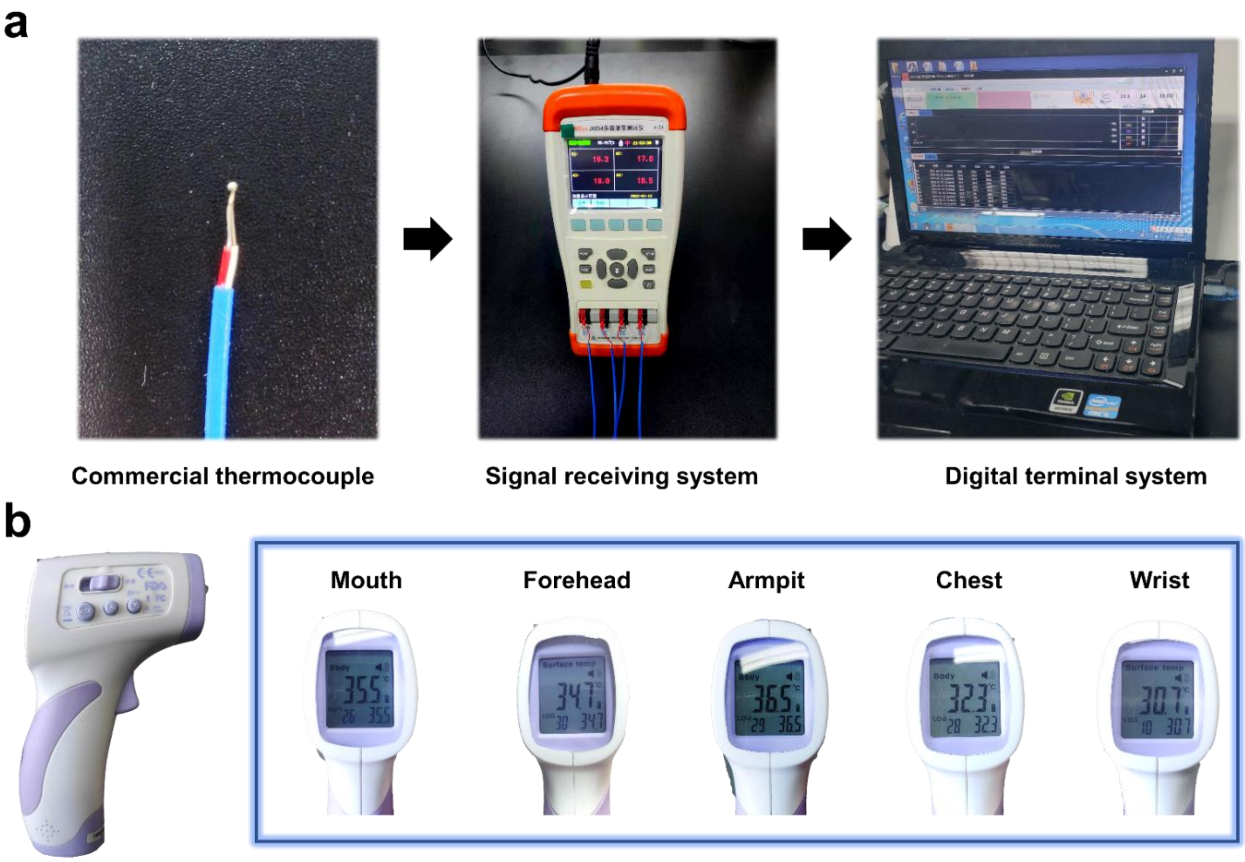

Supplementary Fig. 33. Instant temperature monitoring. a Illustration the ingredients of commercial thermocouple temperature measuring system. **b** Instant temperature monitoring on different body parts by a handheld infrared thermometer.

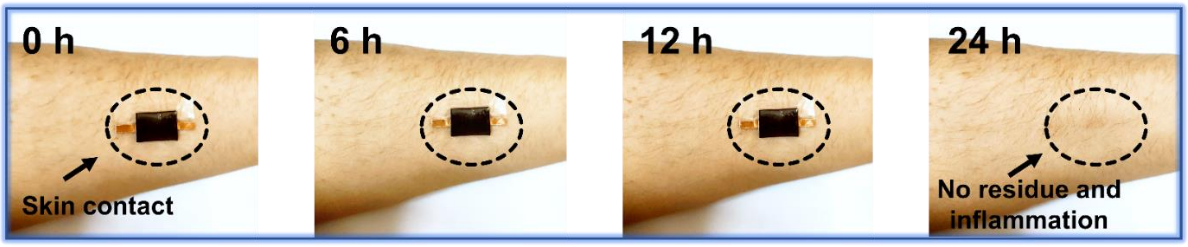

Supplementary Fig. 34. The assembled thermistor elastomer sensor seamlessly contacted to the forearm skin for 24 hours without residue and inflammation after detachment.

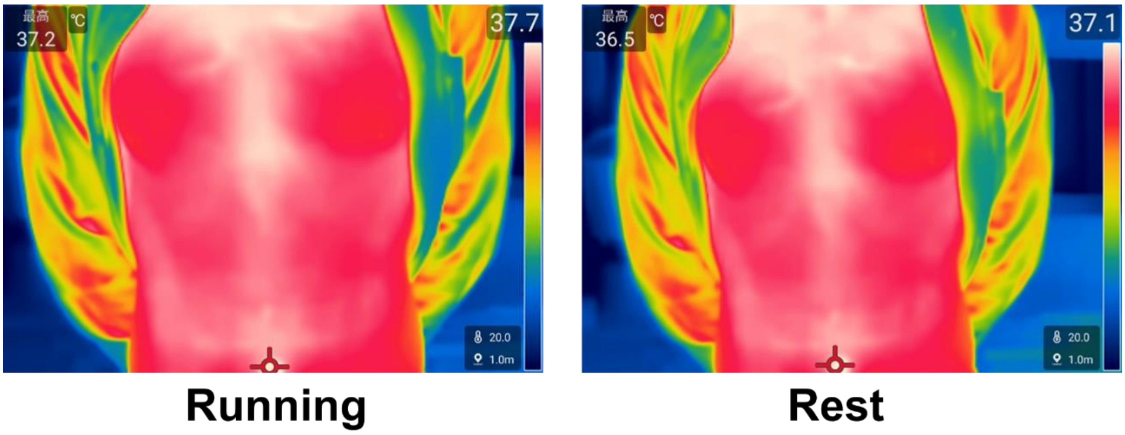

Supplementary Fig. 35. Real-time infrared imaging of a volunteer's trunk during running (37.7 °C) and after running (37.1 °C).

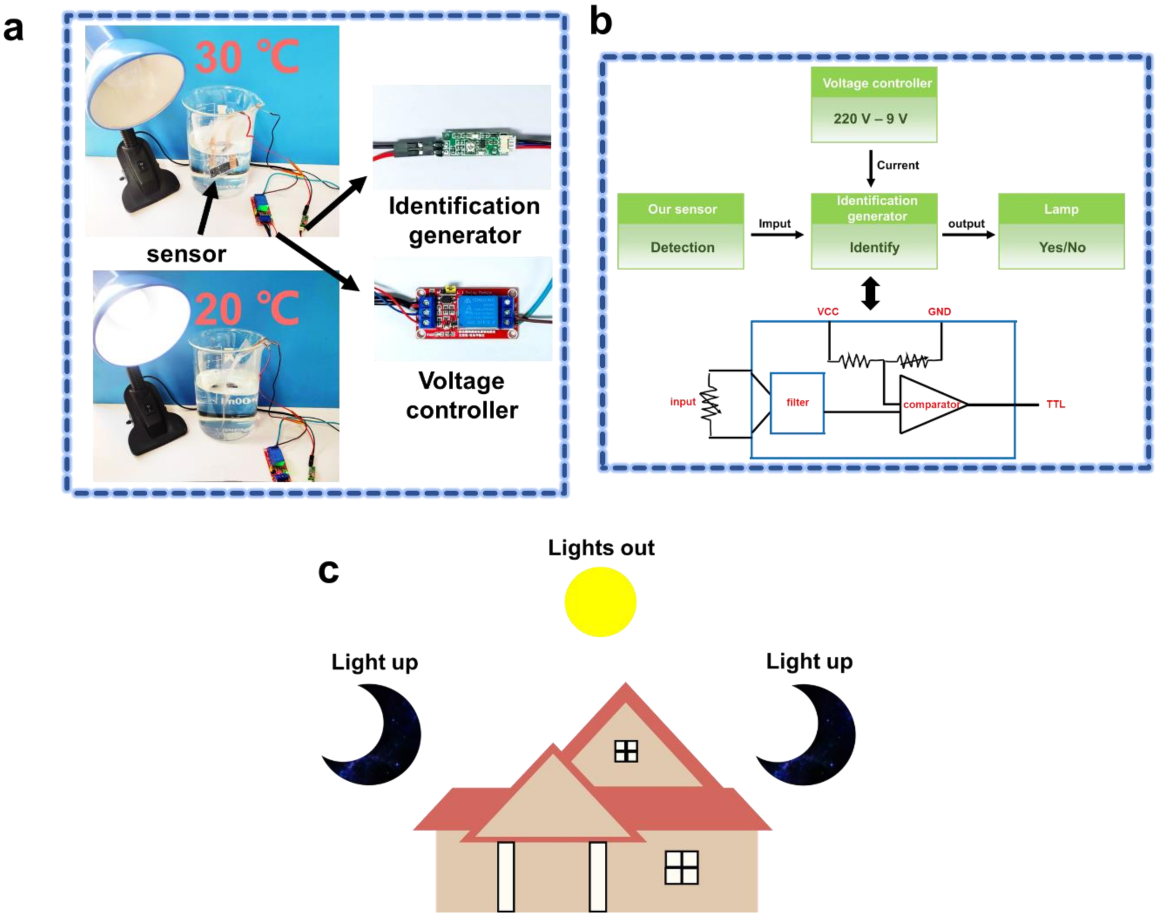

Supplementary Fig. 36. Thermistor sensor control system. a Photos of the assembled thermistor sensor control system realizing the lamp turn-on and turn-off control based on subtle temperature sensitivity mechanism. **b** The logic flow for realizing intelligent temperature sensing. **c** Schematic diagram of lighting up the street lamps in low temperature condition at night.

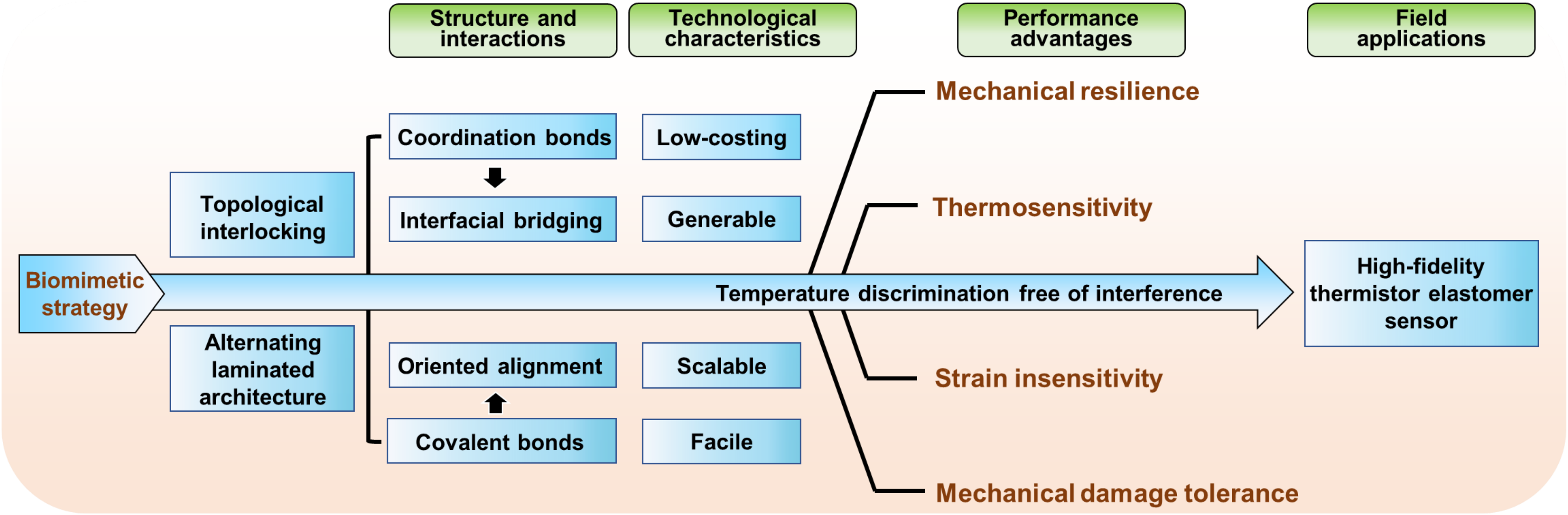

Supplementary Fig. 37. The summary of structural characteristics and beneficial features of the proposed biomimetic laminated strategy.

Supplementary Note 1. Preparation and proposed mechanism of high crystallinity PVA polymer networks.

With respect to the film supporting skeleton, PVA is chosen to ensure structural integrity of the nanocomposite films due to its good water solubility, cost-effective, low-toxicity, and superior mechanical robustness. According to previous reports,[1] the construction of dense polymer networks and achievement of high degree of crystallinity are the major contributing factors to prepare the PVA layers with high mechanical properties and structure stability. Herein, the applying of a strong alkaline hydroxide (6 M) into a dried PVA single layer brings the twofold implications. First, OH^{-} of the alkaline hydroxide attacks the hydroxyl groups of PVA, resulting in disrupted hydrogen bonds and deprotonation of the hydroxyl groups of the PVA chains that described as Eq. S1:

$$R - OH + Na^{+}OH^{-} = R - O^{-}\ Na^{+} + H_2O \qquad (S1)$$

Second, the complexation can be formed between O^{-} group and the free Na^{+}, facilitating the mobility of PVA chains to be aligned and formation of crystalline domains. After adding water to remove Na^{+} ions, the O^{-} became protonated and crystalline domain was permanently stabilized, leading to the PVA polymer networks with high crystallinity.

Supplementary Note 2. Structural optimization towards mechanical performances.

Although the large-size PTF/MXene/Fe composites (sensing layer, 25 × 45 cm^2) can be fabricated via the straightforward interfacial bridging and facile LBL assembly procedure towards the commercial application (Supplementary Fig. 10a), the basic mechanical requirements of FTEE withstanding arbitrary deformations requires further structural optimization for mechanically resilience and robustness, which weighs equal significance compared to the thermosensitivity. For this concern, the influence of the stacked layers number (1, 4, 8, and 16) on mechanical performance is initially discussed owing to its preliminary role in balancing the stiffness and elasticity (Supplementary Fig. 10b), and the sensing layer demonstrated the dramatic improvement in robustness by readily altering the layer number (the corresponding thickness from 0.12 to 0.99 mm) that attained the maximum tensile strain and stress of 1694.8 % and 5.76 MPa, respectively. Considering the excessive layers (thickness increasing) inevitably compromise the mechanical harmony with skin because of the excessive rigidity, the eight-layer of alternating laminated architecture is chosen as the optimized configuration.

Additionally, the uniaxial tensile tests corroborate that the proportion of TOCNF (from 0.25 to 1.25 wt%) is crucial to further optimize the mechanical properties of PTF/MXene composites across a wide range including tensile stress from 3.38 to 6.68 MPa and Young's modulus from 0.11 to 0.39 MPa, respectively (Supplementary Fig. 10c). Simultaneously, the pronounced increasing of elasticity with the addition of Fe(II) is demonstrated for all counterpart samples, convincing the desirable reinforcement effect of the coordination bonds (Supplementary Fig. 10d). In view of the skin-like Young's modulus (0.35 ± 0.032 MPa) and a relatively high toughness (50.62 ± 1.53 MJ m^{-3}), the TOCNF content of 1 wt% is optimal for the subsequent context.

Moreover, the PTF/MXene/Fe composites can be easily elongated to more than 10 times its original length without fracture by uniaxial test, revealing excellent elasticity and stretchability (Supplementary Fig. 10e and 10f). Compared with the PVA composites, the mechanical performances of PTF/MXene/Fe composites were significantly improved, including tensile stress (from 3.38 to 6.68 MPa), elastic modulus (from 0.11 to 0.39 MPa), and toughness (from 28.58 to56.98 MJ m^{-3}). This phenomenon could be mainly attributed to the stiffness of individual TOCNF and dynamic interactions (hydrogen bonds) among TOCNF, MXene nanosheets, and polymer matrix, leading to synergistic effects on the mechanical resilience. Besides, the unique interfacial bridging of coordination bonds between -COOH groups and Fe(II)

contribute to the increasement in both stiffness and toughness without sacrificing extensibility.

Supplementary Note 3. Finite element simulation.

In the finite element simulation, the PTF/MXene/Fe composites with alternating laminated architecture (thickness = 0.6 mm) are modelled as a linear elastic material with Lagrangian formulation with predetermined parameters including density (ρ) = 1.3 g/cm^3, Young's modulus (E) = 0.35 MPa, Poisson ratio (υ) = 0.35. The boundary conditions are set at the bottom of the architecture model, and a gradually increased smooth downward velocity of the model with the final displacement on Z direction of 200 μm within 0.2 s and an average velocity of 1 mm s^{-1}.[2-6]

In order to dynamically manifest the stress distribution in nacre-mimetic architecture during distortion, we developed a 3D finite element model using CINEMA 4D R20 software for the nacre-like structure that duplicated the "brick-and-mortar" arrangement from mollusk shells to achieve the finite element simulation (ABAQUS).With regard to the interface of adjacent layer, we designed the layer volume network combined with random volume growth strategy to mimic the random cross-linking between adjacent layers, thus the adjacent layer possessed random and discrete interface gap as default option in ABAQUS simulation.

Supplementary Table 1. Gibbs free energies of hydration for different metal ions.

Metal ions	ΔG (KJ mol^{-1})
K^{+}	-295
Ni^{2+}	-1980
Mg^{2+}	-1830
Co^{2+}	-1915
Fe^{2+}	-1840
Al^{3+}	-4525
Co^{3+}	-4495
Fe^{3+}	-4265

Supplementary Table 2. Comparison of the thermosensitivity, resolution ratio, operating temperature range, and deformation insensitivity of TES in our work with previous reported thermistor sensors.

Materials	Thermosensitivity (% °C^{-1})	Resolution ratio (°C)	Operating temperature range (°C)	Deformation insensitivity	Refs.
CNT/InGaZnO	0.68	0.3	22.4-40	Yes	8
Electrospun carbon nanofiber	2.44	N/A	25-50	No	9
CNTs/CB/PVA/Gly	0.935	N/A	30-90	No	10
CNT/PEDOT:PSS	0.9	N/A	20-55	No	11
rGO	2.04	0.2	26-101	No	12
rGO/PU	0.55	0.9	0-100	Yes	13
rGO fiber	0.8	0.37	25-50	Yes	14
CVD grown graphene	0.42	N/A	25-50	Yes	15
PBH/rGO	0.3	N/A	20-65	Yes	16
rGO	1.3	0.1	24-45	Yes	17
Pt/constantan alloy	0.024	0.5	0-70	Yes	18
Polypyrrole/Ag NWs	0.086	N/A	17-50	No	19
Ag NFs/Ag NWs	0.03	N/A	30-45	No	20
Ag/Pt NFs/SF	0.205	0.5	20-60	No	21
Gr/SF/Ca (II) ions	2.09	N/A	20-50	No	22
SF/Ca (II) ions	1.9	N/A	30-80	No	23
Au	0.2	0.023	25-50	No	24
SBMA/PEGDA/PDA/Gly	1.33	N/A	0-60	No	25
NaCl/SA/poly acrylic-acrylamide	0.67	N/A	20-65	No	26
CMC/NIPAM	0.925	N/A	20-80	No	27
BP/LEG on SEBS	0.1736	N/A	25-50	Yes	28
PAA/PANI/Gly/Fe^{3+}	1.64	2.7	30-80	Yes	29
PAM-SBMA/TEMPO-CNF-PANI/Gly	2.01	N/A	30-80	Yes	30
PEDOT:PSS/CA NFs/PVA	0.75	3	25-55	No	31
PEDOT:PSS/ Photoactive silk sericin / rGO	0.99	N/A	20-50	No	32
PDMS/PEDOT:PSS/rGO	0.42	N/A	30-55	No	33
Polypyrrole	0.5	N/A	35-95	No	34
Copolymer/NaCl	1.1	0.3	10-85	No	35
MXene/PAA/Gly	0.929	N/A	25-40	No	36
**MXene/PVA/TOCNF/Fe**	**1.27**	**0.3**	**20-80**	**Yes**	**Our work**

"Blue": Carbon-based thermistor sensors. "Purple": Graphene-based thermistor sensors. "Green": Metallic-based thermistor sensors. "Orange": Thermistor sensors prepared via other types of thermosensitive materials. "Gray": Conductive polymer-based thermistor sensors. "Red": MXene nanosheets-based thermistor sensors.

Supplementary Table 3. Comparison of the competitive advantages of the assembled epidermal sensor in this work with previous sensors based on various structural strategies.

Materials	Strategy	Schematic illustration	Thermosensitivity (% °C^{-1})	Resolution ratio (°C)	Mechanical damage endurance	Mechanical durability	Deformation insensitivity	Generality	Refs.
Ni/NiO NP ink	Monolithic structure	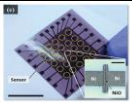	N/A	1	NO	N/A	YES	NO	37
TPU/AgNWs	Kirigami structure	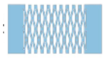	0.7	0.14	NO	N/A	YES	NO	38
rGO/polyurethane	Serpentine geometry structure	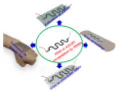	0.8	0.1	NO	N/A	YES	NO	39
Au-doped Si	Serpentine mesh structure	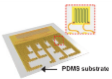	3.15	0.1	NO	5000	YES	NO	40
Au (or Cr/Cu/Al/Ni)/PVA	Matrix array structure	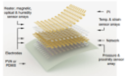	0.228	0.5	NO	N/A	YES	NO	41
Silicone PSA/CFPC/TPU	Anisotropic structure	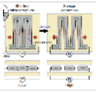	N/A	N/A	YES	1000	YES	YES	42
Cu/Ti/polyimide	Kirigami structure	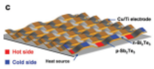	N/A	N/A	NO	1000	YES	NO	43
polyacrylonitrile (PAN)	Electrospun	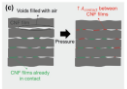	2.44	2.44	NO	3000	YES	YES	44
Cu/PVA	Fiber textile	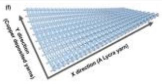	N/A	N/A	NO	3000	YES	NO	45
MXene/TOCNF /PVA	Alternating layered structure	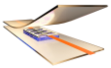	1.32	0.3	YES	20000	YES	YES	Our work

Supplementary References

[1] Li, G. et al, 2D Titanium Carbide (MXene) Based Films: Expanding the Frontier of Functional Film Materials. *Adv. Funct. Mater*. **31**, 2105043 (2021).

[2] Wang, T. et al, Mechanically Durable Memristor Arrays Based on a Discrete Structure Design. *Adv. Mater.* **34**, 2106212 (2022).

[3] Wu, k. et al, A Prestressing Strategy Enabled Synergistic Energy-Dissipation in Impact-Resistant Nacre-Like Structures. *Adv. Sci.* **9**, 2104867 (2022).

[4] Guo, Z. et al, Kirigami-Based Stretchable, Deformable, Ultralight Thin-Film Thermoelectric Generator for BodyNET Application. *Adv. Energy Mater.* **12**, 2102993 (2021).

[5] Yang, T. et al, Hierarchically Microstructure-Bioinspired Flexible Piezoresistive Bioelectronics. *ACS Nano* **15**, 11555 (2021).

[6] Chai, S. S., Zan, G.T., Dong, K. Z., Wu, T., Wu, Q.S. Approaching Superfoldable Thickness-Limit Carbon Nanofiber Membranes Transformed from Water-Soluble PVA. *Nano Lett.* **21**, 8831(2021).

[7] Darabi, M. A. et al, An Alkaline Based Method for Generating Crystalline, Strong, and Shape Memory Polyvinyl Alcohol Biomaterials. *Adv. Sci.* **7**, 1902740 (2020).

[8] Honda, W. et al. High-performance, mechanically flexible, and vertically integrated 3D carbon nanotube and InGaZnO complementary circuits with a temperature sensor. *Adv. Mater.* **27**, 4674 (2015).

[9] Lee, J. H. et al. Rational Design of All Resistive Multifunctional Sensors with Stimulus Discriminability. *Adv. Funct. Mater.* **32**, 2107570 (2021).

[10]Gu, J. et al. Multifunctional Poly(vinyl alcohol) Nanocomposite Organohydrogel for Flexible Strain and Temperature Sensor. *ACS Appl. Mater. Interfaces* **12**, 40815 (2020).

[11] Kanao, K. et al. Highly selective flexible tactile strain and temperature sensors against substrate bending for an artificial skin. *RSC Adv*. **5**, 30170 (2015).

[12] Wu, J. et al. Self-Calibrated, Sensitive, and Flexible Temperature Sensor Based on 3D Chemically Modified Graphene Hydrogel. *Adv. Electron. Mater.* **7**, 2001084 (2021).

[13]Trung, T. Q., Ramasundaram, S., Hwang, B. U., Lee, N. E. An all-elastomeric transparent and stretchable temperature sensor for body-attachable wearable electronics. *Adv. Mater.* **28**, 502 (2016).

[14] Trung, T. Q. et al. A stretchable strain-insensitive temperature sensor based on free-standing elastomeric composite fibers for on-body monitoring of skin temperature. *ACS Appl. Mater. Interfaces* **11**, 2317 (2019).

[15]Kabiri Ameri, S. et al. Graphene electronic tattoo sensors. *ACS Nano* **11**, 7634 (2017).

[16]Chen, S., Chen, Y., Li, D., Xu, Y., Xu, F. Flexible and Sensitivity-Adjustable Pressure Sensors Based on Carbonized Bacterial Nanocellulose/Wood-Derived Cellulose Nanofibril Composite Aerogels. *ACS Appl. Mater. Interfaces* **13**, 7, 8754-8763 (2021).

[17] Liu, Q. et al. A High-Performances Flexible Temperature Sensor Composed of Polyethyleneimine/Reduced Graphene Oxide Bilayer for Real-Time Monitoring. *Adv.*

Mater. Technol. **4**, 1800594 (2019).
[18] Hua, Q. et al. Skin-inspired highly stretchable and conformable matrix networks for multifunctional sensing. *Nat. Commun.* **9**, 244 (2018).
[19] Bang, J. et al. Highly Sensitive Temperature Sensor: Ligand-Treated Ag Nanocrystal Thin Films on PDMS with Thermal Expansion Strategy. *Adv. Funct. Mater.* **29**, 1903047 (2019).
[20] An, B. W., Heo, S., Ji, S., Bien, F., Park, J. U. Transparent and flexible fingerprint sensor array with multiplexed detection of tactile pressure and skin temperature. *Nat. Commun.* **9**, 2458 (2018).
[21] Huang, J. N. et al. Stretchable and Heat-Resistant Protein-Based Electronic Skin for Human Thermoregulation. *Adv. Funct. Mater.* **30**, 1910547(2020).
[22] Wang, Q. et al. Self-healable multifunctional electronic tattoos based on silk and graphene. *Adv. Funct. Mater.* **29**, 1808695 (2019).
[23] Liu, J. et al. Intelligent silk fibroin ionotronic skin for temperature sensing. *Adv. Mater. Technol.* **5**, 2000430 (2020).
[24] He, Y. et al. Coiled Fiber-Shaped Stretchable Thermal Sensors for Wearable Electronics. *Adv. Mater. Technol.* **1**, 1600170 (2016).
[25] Webb, R. C. et al. Ultrathin conformal devices for precise and continuous thermal characterization of human skin. *Nat. Mater.* **12**, 938 (2013).
[26] Huang, H. et al. Super-stretchable, elastic and recoverable ionic conductive hydrogel for wireless wearable, stretchable sensor. *J. Mater. Chem. A.* **8**,10291-10300 (2020).
[27] Chen, Z. et al. Multiple-stimuli-responsive and cellulose conductive ionic hydrogel for smart wearable devices and thermal actuators. *ACS Appl. Mater. Interfaces* **13**, 1353 (2021).
[28] Chhetry, A. et al. Black Graphene Heterostructure-Based Temperature-Strain Hybridized Sensor for Electronic-Skin Applications. *Adv. Funct. Mater.* **31**, 2007661 (2020).
[29] Ge, G. et al. Muscle-Inspired Self-Healing Hydrogels for Strain and Temperature Sensor. *ACS Nano* **14**, 218 (2020).
[30] Hao, S., Meng, L., Fu, Q., Xu, F., Yang, J. Low-Temperature tolerance and conformal adhesion zwitterionic hydrogels as electronic skin for strain and temperature responsiveness. *Chem. Eng. J.* **431**, 133782 (2022).
[31] Yue, O. et al. Spider-Web and Ant-Tentacle Doubly Bio-Inspired Multifunctional Self-Powered Electronic Skin with Hierarchical Nanostructure. *Adv. Sci.* **8**, 2004377 (2021).
[32] Pradhan, S., Yadavalli, V. K. Photolithographically Printed Flexible Silk/PEDOT:PSS Temperature Sensors. *ACS Appl. Electron. Mater.* **3**, 21-29 (2020).
[33] Yu, Y., Peng, S., Blanloeuil, P., Wu, S., Wang, C. H. Wearable temperature sensors with enhanced sensitivity by engineering microcrack morphology in PEDOT: PSS–PDMS sensors. *ACS Appl. Mater. Interfaces* **12**, 36578 (2020).
[34] He, Y. et al. Coiled Fiber-Shaped Stretchable Thermal Sensors for Wearable Electronics. *Adv. Mater. Technol.* **1**, 1600170 (2016).
[35] Lei, Z. et al. A supramolecular biomimetic skin combining a wide spectrum of

mechanical properties and multiple sensory capabilities. *Nat. Commun.* **9**, 1134 (2018).
[36] Ge, G. et al. N$Ti_3C_2T_x$ MXene-Activated Fast Gelation of Stretchable and Self-Healing Hydrogels: A Molecular Approach. *ACS Nano* **15**, 2698 (2021).
[37] Shin, J. et al. Sensitive Wearable Temperature Sensor with Seamless Monolithic Integration. *Adv. Mater.* **32**, 1905527 (2020).
[38] Yu, Y., Peng, S., Blanloeuil, P., Wu, S., Wang, C. H. Wearable temperature sensors with enhanced sensitivity by engineering microcrack morphology in PEDOT: PSS–PDMS sensors. *ACS Appl. Mater. Interfaces* **12**, 36578 (2020).
[39] Trung, T. Q. et al. A stretchable strain-insensitive temperature sensor based on free-standing elastomeric composite fibers for on-body monitoring of skin temperature. *ACS Appl. Mater. Interfaces* **11**, 2317 (2019).
[40] Sang, M. et al. Ultrahigh Sensitive Au-Doped Silicon Nanomembrane Based Wearable Sensor Arrays for Continuous Skin Temperature Monitoring with High Precision. *Adv. Mater.* **34**, 2105865 (2022).
[41] Hua, Q. et al. Skin-inspired highly stretchable and conformable matrix networks for multifunctional sensing. *Nat. Commun.* **9**, 244 (2018).
[42] Araromi, O. A. et al. Ultra-sensitive and resilient compliant strain gauges for soft machines. *Nature* **587**, 219 (2020).
[43] Guo, Z. et al. Kirigami-Based Stretchable, Deformable, Ultralight Thin-Film Thermoelectric Generator for BodyNET Application. *Adv. Energy Mater.* **12**, 2102993 (2021).
[44] Lee, J. H. et al. Rational Design of All Resistive Multifunctional Sensors with Stimulus Discriminability. *Adv. Funct. Mater.* **32**, 2107570 (2021).
[45] Li, X. et al. MXene chemistry, electrochemistry and energy storage applications. *Nat. Rev. Chem*. **6**,389-404 (2022).
