# Supplementary material for: A biomimetic laminated strategy enabled strain-interference free and durable flexible thermistor electronics
Source: Nat Commun. 2022 Oct 29;13:6472. doi: 10.1038/s41467-022-34168-x (PMC9617538; doi:10.1038/s41467-022-34168-x)
Supplement: Supplementary file 3 — Description of Additional Supplementary Files [file 41467_2022_34168_MOESM3_ESM.pdf]

Description of Additional Supplementary Files

File name: Supplementary Movie 1
Description: The in-plane stress dispersion of the PTF/MXene/Fe composites with alternating laminated architecture.

File name: Supplementary Movie 2
Description: The fast gelation of TOCNF/MXene solutions by Fe(Ⅱ) cross-linking.

File name: Supplementary Movie 3
Description: The sensitive temperature discrimination demonstration as the large and instant I-T profile fluctuations.

File name: Supplementary Movie 4
Description: The high-fidelity temperature discrimination free of strain induced signal fusion, which can withstand consecutive hammering for restraining the signal fluctuation.

File ame: Supplementary Movie 5
Description: Demonstration of lamp turn-on and turn-off control device based on temperature gradient by immersing the assembled TES to water-filled beaks with different temperatures.
